# Tethering Cells via Enzymatic Oxidative Crosslinking Enables Mechanotransduction in Non‐Cell‐Adhesive Materials

**DOI:** 10.1002/adma.202102660

**Published:** 2021-09-03

**Authors:** Tom Kamperman, Sieger Henke, João F. Crispim, Niels G. A. Willemen, Pieter J. Dijkstra, Wooje Lee, Herman L. Offerhaus, Martin Neubauer, Alexandra M. Smink, Paul de Vos, Bart J. de Haan, Marcel Karperien, Su Ryon Shin, Jeroen Leijten

**Affiliations:** ^1^ Department of Developmental BioEngineering Faculty of Science and Technology Technical Medical Centre University of Twente Drienerlolaan 5 Enschede 7522NB The Netherlands; ^2^ Division of Engineering in Medicine Brigham and Women's Hospital Harvard Medical School 65 Landsdowne Street Cambridge MA 02139 USA; ^3^ Optical Sciences MESA+ Institute for Nanotechnology University of Twente Drienerlolaan 5 Enschede 7522NB The Netherlands; ^4^ Physical Chemistry II University of Bayreuth Universitätsstrasse 30 D‐95447 Bayreuth Germany; ^5^ Department of Pathology and Medical Biology Section of Immunoendocrinology University of Groningen University Medical Center Groningen Hanzeplein 1 (EA11) Groningen 9713 GZ The Netherlands

**Keywords:** adhesomes, biomechanics, cell volume, inflammation, lineage commitment, single‐cell analysis, stem cell microniches

## Abstract

Cell–matrix interactions govern cell behavior and tissue function by facilitating transduction of biomechanical cues. Engineered tissues often incorporate these interactions by employing cell‐adhesive materials. However, using constitutively active cell‐adhesive materials impedes control over cell fate and elicits inflammatory responses upon implantation. Here, an alternative cell–material interaction strategy that provides mechanotransducive properties via discrete inducible on‐cell crosslinking (DOCKING) of materials, including those that are inherently non‐cell‐adhesive, is introduced. Specifically, tyramine‐functionalized materials are tethered to tyrosines that are naturally present in extracellular protein domains via enzyme‐mediated oxidative crosslinking. Temporal control over the stiffness of on‐cell tethered 3D microniches reveals that DOCKING uniquely enables lineage programming of stem cells by targeting adhesome‐related mechanotransduction pathways acting independently of cell volume changes and spreading. In short, DOCKING represents a bioinspired and cytocompatible cell‐tethering strategy that offers new routes to study and engineer cell–material interactions, thereby advancing applications ranging from drug delivery, to cell‐based therapy, and cultured meat.

## Graphical abstract

Cells need to sense their environment in order to survive and function. In this study an enzymatic crosslinking strategy that can tether non‐cell‐adhesive materials to cells is presented. By targeting the cellular adhesome, this cell–material tethering method enables cell‐volume‐independent lineage programming of stem cells in engineered 3D microniches with controlled stiffness.

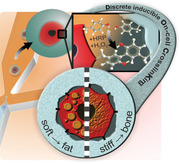

## 1 Introduction

Cell–matrix interactions transduce biomechanical cues, which govern numerous cellular functions including migration, proliferation, apoptosis, metabolism, and differentiation.^[^ ^1^, ^2^ ^]^ However, the variety of strategies to control cell–biomaterial interactions able to transmit mechanical cues from the microenvironment to the cells within engineered tissue constructs has remained limited and near‐exclusively relied on cell adhesion. Biomaterials are typically endowed with bioligands that bind to cell adhesion molecules (CAMs; e.g., integrins, cadherins, and selectins). The integrin‐binding tripeptide arginine‐glycine‐aspartic acid (RGD)^[^ ^3^ ^]^ has been most commonly used as cell‐adhesive moiety, but also numerous alternative cell‐adhesive nucleotides,^[^ ^4^ ^]^ peptides,^[^ ^5^ ^]^ and proteins including antibodies^[^ ^6^ ^]^ and amyloid‐like lysozyme^[^ ^7^ ^]^ have been explored. However, the constitutively active binding nature of these cell‐adhesive bioligands has been associated with adverse effects such as increased fibrous capsule formation and chronic inflammation upon implantation.^[^ ^8^, ^9^ ^]^ Dynamic material modification strategies that display cell‐adhesive bioligands with precise spatial and temporal control have been developed to address this challenge. However, once activated, their cell‐adhesive properties are continuously active and can only be annihilated using non‐physiological artificial external triggers such as electrical potential, UV light, or competing binders,^[^ ^8^, ^10^ ^]^ which challenges clinical translation. We identified the need for an alternative cell–material interaction strategy that offers mechanotransducive properties to intrinsically non‐cell‐adhesive biomaterials via a cytocompatible, physiological, and clinically translatable approach.

Inspired by nature, we aimed to directly tether cells to materials that are intrinsically bio‐inert and do not present any constitutively active bioligands via a discrete (i.e., temporally controlled) inducible enzymatic crosslinking reaction. Enzyme‐mediated oxidative crosslinking using peroxidases is naturally observed in physiological processes where it provides, for example, structural stability and protection on protein, tissue, and even organ(ism) levels through the formation of dityrosines.^[^ ^11^ ^]^ Moreover, enzyme‐mediated oxidative crosslinking of phenolic moieties has been successfully explored in tyramide signal amplification,^[^ ^12^ ^]^ peroxidase‐mediated proteomic mapping strategies within cells,^[^ ^13^ ^]^ and tissue engineering strategies.^[^ ^14^, ^15^ ^]^ Despite the abundant presence of tyrosines in ECM proteins and the extracellular domain of transmembrane proteins,^[^ ^16^ ^]^ crosslinking of phenolic compounds to tether biomaterials directly onto living cells has not yet been explored.

Here, we report on the discrete inducible on‐cell crosslinking (DOCKING) of non‐cell‐adhesive biomaterials onto cells via oxidative crosslinking of phenolic moieties. Specifically, we demonstrated that tyramine‐functionalized dextran (Dex‐TA) could be enzymatically crosslinked with tyrosine‐rich extracellularly presented proteins such as fibronectin, thereby forming hydrogel microniches that are tethered to cells via a mechanism that does not depend on cell adhesion. The intrinsically bio‐inert and non‐cell‐adhesive Dex‐TA hydrogel elicited a reduced inflammatory response upon implantation in mice as compared to its RGD‐modified cell‐adhesive counterpart. Importantly, enzyme‐mediated oxidative crosslinking enabled the controlled mechanotransduction from non‐cell‐adhesive Dex‐TA hydrogel to encapsulated cells via cell tethering. As a proof‐of‐concept, we leveraged an advanced microfluidic system to tether individual mesenchymal stem/stromal cells (MSCs) within micrometer‐sized hydrogel matrices (i.e., microgels) using DOCKING to control MSC function and fate at single‐cell resolution. Through timed modulation of microgel stiffness, we could program the lineage commitment of microencapsulated stem cells, thereby indicating DOCKING‐mediated mechanotransduction in a bioligand‐free material. 3D‐tethering MSCs in non‐degradable and non‐cell‐adhesive hydrogel revealed that mechanotransduction and lineage programming can occur independent of cell volume changes and spreading. As cell–material interactions are instrumental in guiding tissue development, organ homeostasis, disease progression, and repair processes, DOCKING represents a unique tool for optimizing tissue engineering applications, such as regenerative medicine, cultured meat, and organ‐on‐chip platforms.

## 2 Results and Discussion

### 2.1 Discrete Inducible Tethering of Cells and Non‐Cell‐Adhesive Materials via Enzyme‐Mediated Oxidative Crosslinking

**Figure 1:**
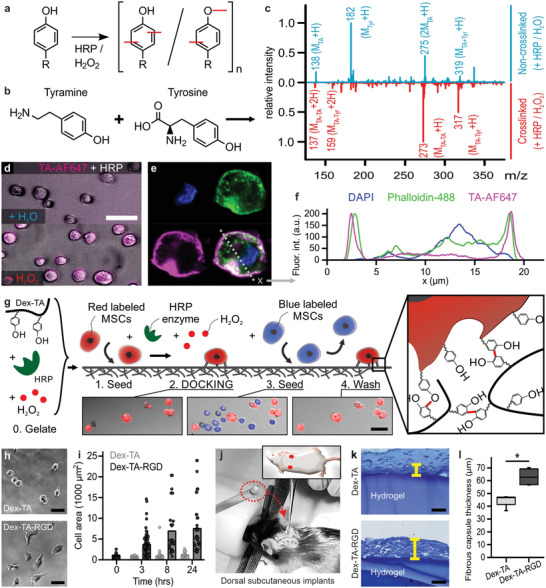
Discrete inducible tethering of cells and non‐cell‐adhesive materials via enzyme‐mediated oxidative crosslinking. a) Phenolic moieties can be enzymatically coupled and oligomerized using horseradish peroxidase (HRP) and hydrogen peroxide (H_2_O_2_) via the formation of C—C and C—O bonds. b,c) ESI‐MS confirmed the enzymatic crosslinking (i.e., red peaks) of tyramine to tyramine ([M_TA–TA_ + H]^+^: 273, [M_TA–TA_ + 2H]^2+^: 137) and tyramine to tyrosine ([M_TA–Tyr_ + H]^+^: 317, [M_TA–Tyr_ + 2H]^2+^: 159). The blue plot indicates the control experiment (i.e., with dH_2_O instead of H_2_O_2_). d) Enzyme‐mediated crosslinking could also be leveraged to couple fluorescently labeled tyramine (TA‐AF647, magenta) directly onto cells, e,f) thereby predominantly staining pericellularly as shown using confocal microscopy. Cells were stained with phalloidin (green) and DAPI (blue). g) Endowing dextran with tyramine moieties enabled the formation of Dex‐TA hydrogel substrates (i.e., “0. Gelate”), onto which (red labeled) cells could be seeded (i.e., “1. Seed”) and tethered using an enzymatic post‐cure (i.e., “2. DOCKING”). In contrast, (blue labeled) cells that were seeded (i.e., “3. Seed”) but not tethered to the same Dex‐TA substrate were easily washed away (i.e., 4. Wash'). h,i) MSCs adhered and spread on Dex‐TA‐RGD, but not on Dex‐TA substrates. j–l) Dorsal subcutaneous implantation of Dex‐TA hydrogel disks in C57BL/6 mice revealed significantly less fibrotic capsule formation as compared to disks made of Dex‐TA‐RGD. The yellow lines indicate representative fibrotic capsule thickness measurements of Toluidine Blue stained sections. Boxes indicate 25–75 percentiles, lines indicate medians, whiskers indicate min–max, *n* = 4, significance is indicated (^*^ *p* <  0.05; Mann–Whitney). The scale bars indicate 50 µm.

We set out to exploit a biomaterial that could be tethered to cells via an on‐demand inducible and cytocompatible crosslinking reaction. Inspired by nature and our previous work on self‐attaching hydrogels,^[^ ^14^ ^]^ we selected a strategy based on enzymatic crosslinking of macromolecules containing phenolic moieties. Coupling and oligomerization of conjugated phenolic moieties via C—C and C—O bond formation can be carried out using a catalyst (e.g., horseradish peroxidase (HRP)) and an oxidizer (e.g., hydrogen peroxide (H_2_O_2_); **Figure** **1**a).^[^ ^17^ ^]^ To demonstrate that tyramines can crosslink with tyrosines, a model experiment was performed in which tyramine and tyrosine solutions were mixed and reacted using HRP and H_2_O_2_ (Figure 1b). Electrospray ionization mass spectrometry (ESI‐MS) confirmed the presence of coupled tyramine–tyramine and tyramine–tyrosine products in the HRP‐catalyzed reaction with H_2_O_2_ while no crosslinking of tyramine and tyrosine was observed in the absence of H_2_O_2_ (Figure 1c). As extracellular domains contain a significant amount of tyrosines, we reasoned that cells could be decorated with a range of entities via phenolic crosslinking. Indeed, different mammalian cell types including MSCs were labeled by tethering tyramine‐functionalized fluorophores (i.e., TA‐AF647) to cells using HRP and H_2_O_2_ (Figure 1d; Figure S1, Supporting Information). Confocal fluorescence microscopy revealed that TA‐AF647 was predominantly located extracellularly in the direct vicinity of the plasma membrane (Figure 1e,f). Together, this proved that DOCKING can be used to pericellularly decorate living cells in a fast, efficient, and cytocompatible manner.

We next aimed to leverage DOCKING for tethering (bioligand‐free) biomaterials to cells and provide biomechanical cues to cells in a manner that is independent of conventional cell adhesion. To this end, we conjugated tyramines to a dextran polymer backbone, which is a natural and cytocompatible, yet non‐cell‐adhesive polymer,^[^ ^18^, ^19^ ^]^ resulting in an injectable biomaterial that could potentially crosslink to tyrosine residues on cells’ membranes (Figure S2, Supporting Information). Dex‐TA disks formed via enzymatic crosslinking still contained tyramines that were available for further functionalization with phenolic moieties by repeating the enzymatic crosslinking procedure (Figure S3, Supporting Information). To demonstrate the direct tethering of Dex‐TA to cells, fluorescently labeled MSCs were seeded on top of preformed Dex‐TA hydrogel disks and underwent the enzymatic crosslinking reaction (Figure 1g). DOCKING rapidly and securely bound cells onto the otherwise non‐cell‐adhesive hydrogel surface; vigorous washing did not remove the cells from the hydrogel surface. In contrast, MSCs that were not tethered to the hydrogel via enzymatic oxidative crosslinking were instantly removed by mild washing. Enzymatic digestion of extracellular peptides using the serine protease trypsin efficiently released tethered cells in a cytocompatible manner (Figure S4, Supporting Information). This indicated that Dex‐TA was predominantly crosslinked to pericellular proteins that were subjectable to enzymatic digestion. To confirm that the cell–material interaction was specifically induced via DOCKING and not dynamic adaptation of cells owed to their altered microenvironment, we confirmed our findings by tethering formalin‐fixated cells to Dex‐TA (Figure S5, Supporting Information).

We then investigated whether DOCKING could affect cell receptor functioning. To this end, we selected C2C12 cells that were stably transfected with a reporter plasmid consisting of bone morphogenetic protein (BMP)‐responsive elements from the Id1 promoter fused to a luciferase reporter gene (C2C12‐BRE‐Luc).^[^ ^20^ ^]^ These cells express various BMP type 1 and 2 receptors that are all characterized by an extracellular topological domain containing multiple tyrosine residues. Tethering Dex‐TA onto C2C12‐BRE‐Luc cells using the DOCKING protocol did not negatively impact the response of reporter cells to BMP2, which suggested that DOCKING has, at least for canonical BMP signaling, no impairing effect on tyrosine‐containing cell surface receptors (Figure S6, Supporting Information).

As it has been reported that an intrinsically non‐cell‐adhesive hydrogel elicits a smaller chronic inflammatory response as compared to a similar implant functionalized with RGD peptides,^[^ ^8^ ^]^ we hypothesized that DOCKING should also associate with reduced implant fibrosis. To this end, we prepared 2 × 5 mm sized disks consisting of Dex‐TA or Dex‐TA‐RGD. Culturing MSCs on the surface of the hydrogel disks confirmed that (in the absence of DOCKING) MSCs could only attach and spread on Dex‐TA‐RGD, but not on Dex‐TA (Figure 1h,i; Figure S7, Supporting Information). Subcutaneously implanted Dex‐TA disks in C57BL/6 mice showed significantly less fibrotic capsule formation as compared to Dex‐TA‐RGD 4 weeks post implantation. Identifying and quantifying the relative abundance of inflammatory cells also indicated that more fibroblasts were present around the Dex‐TA‐RGD disks (Figure S8, Supporting Information). Together, these results suggested that DOCKING offers a potent strategy to tether cells to non‐cell‐adhesive materials in a manner that associates with a reduced host immune response when implanted (Figure 1j–l).

### 2.2 DOCKING Enables Engineering of Mechanically Instructive Stem Cell Niches

**Figure 2:**
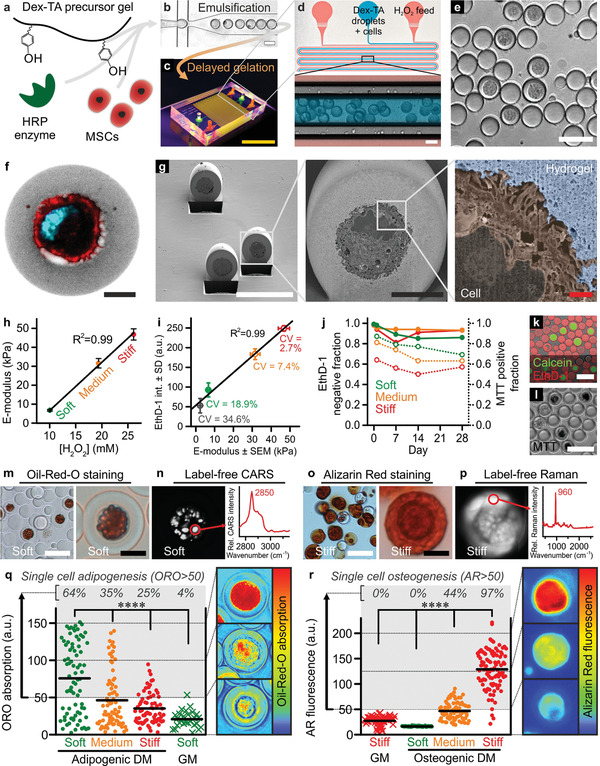
DOCKING enables engineering of mechanically instructive stem cell niches. a) Dex‐TA, HRP, and MSCs were mixed, b) emulsified using droplet microfluidics, and c,d) reacted using a diffusion‐based H_2_O_2_ supplementation platform into e) single‐cell‐laden microgels that could be retrieved by breaking the emulsion. f) Confocal microscopy and g) FIB/SEM (pseudo‐colored) revealed successful encapsulation of individual MSCs in the center of Dex‐TA microgels and suggested that DOCKING resulted in the attachment of cells to the Dex‐TA microgel interior. A single‐cell microgel (f) was labeled with FITC (grey), phalloidin (red), and DAPI (cyan). h) The *E*‐modulus of microgels could be tuned between ≈5 and 50 kPa and linearly depended on the concentration of H_2_O_2_. Error bars indicate ± standard error, *n* ≥ 32. i) Besides staining nuclei of dead cells, EthD‐1 also stained Dex‐TA and its intensity linearly (*R* ^2^ = 0.99) correlated to the microgel *E*‐modulus. Coefficients of variation (CV = standard deviation/average) of various microgel populations including soft, medium, and stiff ones were determined as a relative measure for inter‐microgel variation within populations. The error bars indicate ± standard error, *n* ≥ 32, and ± standard deviation, *n* ≥ 97. j–l) The viable (closed circles) and metabolically active (open circles) cell fractions of in vitro cultured single‐MSC‐laden microgels were determined using live/dead (k) and MTT staining (l). Datapoints indicate average, *n* ≳ 100. m) In soft Dex‐TA microgels, adipogenic differentiation after 4 weeks of culture in adipogenic differentiation medium (DM) was confirmed using Oil‐Red‐O (ORO) staining and n) label‐free detection of lipids using hyperspectral coherent anti‐Stokes Raman scattering (CARS; characteristic lipid peak at 2850 cm^−1^). o) In stiff Dex‐TA microgels, osteogenic differentiation after 4 weeks of culture in osteogenic differentiation medium was confirmed using Alizarin Red (AR) staining and p) label‐free detection of calcium phosphates using hyperspectral spontaneous Raman (characteristic phosphate peak at 960 cm^−1^). q,r) Quantification of the per‐cell adipogenic (q) and osteogenic (r) differentiation as a function of microgel stiffness and culture medium. “GM” indicates growth medium, lines indicate means, *n* ≥ 27 (q), *n* ≥ 55 (r), significance is indicated (^****^ *p* < 0.0001, Kruskal–Wallis analysis of variance (ANOVA), validated with Mann–Whitney individual sample comparison). The yellow scale bar indicates 1 cm, the white scale bars indicate 50 µm, the black scale bars indicate 10 µm, and the red scale bar indicates 1 µm.

We then investigated whether DOCKING could act as a novel bioengineering strategy to convey mechanically instructive stimulation to cells. To this end, a thin conformal Dex‐TA coating was tethered onto individual MSCs using a microfluidic droplet generation platform that we recently developed (**Figure** **2**a–e; Figure S9, Supporting Information).^[^ ^21^ ^]^ The resulting single‐cell microgels offer highly controlled experimental conditions to study cell–material interactions as interference from neighboring cells’ activities including direct cell–cell interactions, paracrine signaling, and ECM deposition is minimized, while yielding single‐cell‐resolution data. Furthermore, the relative small size and high surface‐to‐volume ratio of such single‐cell microgels facilitates their manipulation, culture, and high‐resolution (optical) analysis.^[^ ^22^ ^]^ In accordance with literature, the encapsulated cell fraction closely followed the Poisson distribution (Figure S10, Supporting Information).^[^ ^23^ ^]^ The retrieved cell‐laden microgels (27 ± 2 µm) were monodisperse in size with a coefficient of variation (CV) of ≈7.5% and composed of individual MSCs (18 ± 4 µm) that were conformally coated by a ≈5 µm thin Dex‐TA hydrogel layer (Figure S10, Supporting Information). Although Dex‐TA microgels did not possess intrinsic cell binding capacity (Figure S11, Supporting Information), high‐resolution confocal imaging of single‐cell‐laden microgels suggested that the cell's plasma membrane was attached to the microgel's interior surface at multiple locations (Figure 2f). This observation was corroborated by scanning electron microscopy images of microgels that were prepared using extremely thin layer plastification and dissected using focused ion beam milling (i.e., FIB/SEM; Figure 2g; Figure S12, Supporting Information).^[^ ^24^ ^]^

To determine if cell–material tethering via DOCKING enabled mechanotransduction, we produced single‐cell microniches with tunable stiffness. Advantageously, the diffusion‐based microfluidic crosslinker platform offered high‐fidelity control over the amount of supplemented H_2_O_2_ (Figure S13, Supporting Information), which linearly (*R* ^2^ = 0.99) correlated with microgel stiffness as measured using nano‐indentation (Figure 2h).^[^ ^25^ ^]^ By controlling crosslinking density, we reproducibly produced soft, medium, and stiff microgels with E‐moduli of 6.7 ± 0.4, 31.5 ± 2.5, and 46.8 ± 3.0 kPa, respectively. We then used EthD‐1 staining to study the intra‐ and inter‐network homogeneity of Dex‐TA microgels, as the fluorescence intensity linearly (*R* ^2^ = 0.99) correlated with the microgels’ stiffness (Figure 2i; Figure S14, Supporting Information). High‐resolution confocal visualization confirmed that cell‐laden microniches were homogeneously crosslinked, indicating that encapsulated cells were exposed to similar microelasticities as measured on the material's surface using nano‐indentation (Figure S14, Supporting Information). Furthermore, the relative distribution (i.e., CV) of crosslinking densities between microgels was 18.9%, 7.4%, and 2.7% for soft, medium, and stiff microgels, respectively, which is much smaller than the relative heterogeneity of cellular mechanical properties as observed in clonal MSC populations (i.e., ≈25% to ≈60%).^[^ ^26^ ^]^ Therefore, the relatively homogeneously crosslinked microgels provided well‐controlled mechanical properties, which facilitated the study of biomechanical cell programming by on‐cell tethered microniches.

Short‐ and long‐term effects of single‐cell DOCKING on cell survival in microgels with distinct stiffnesses were assessed using live/dead and metabolic assays. After 28 days of in vitro culture, ≈90% of all encapsulated (i.e., soft, medium, and stiff) MSCs were still viable (Figure 2j,k). The metabolic activity of MSCs inversely correlated with microgel stiffness, which suggested that MSCs sensed their microniche stiffness (Figure 2j,l); changes in matrix stiffness and cell metabolism are known to correlate.^[^ ^27^ ^]^ Although cultured in proliferation medium, we did not observe mitosis of microencapsulated MSCs, which was in line with previous observations of MSCs cultured in confined 3D microniches^[^ ^28^ ^]^ and could be explained by a specific target size presumably needed for cells to enter the synthesis phase (S‐phase).^[^ ^29^ ^]^ However, releasing MSCs from Dex‐TA microgels using dextranase confirmed that DOCKING had no detrimental effect on the cells’ innate binding and proliferation capacity (Figure S15, Supporting Information). Together, this proved that the microfluidic encapsulation procedure and DOCKING‐mediated tethering of Dex‐TA to cells were cytocompatible and yielded mechanically tunable microniches that enabled the study of short‐ and long‐term biomechanical cell programming by on‐cell tethered material.

MSCs were individually encapsulated in Dex‐TA microgels using DOCKING and differentiated into the adipogenic and osteogenic lineages, which was visualized using both conventional histological stains and label‐free analyses (Figure 2m–p; Figure S16, Supporting Information). Quantification of intracellular lipid and extracellular calcified matrix deposition as proxies for adipogenesis and osteogenesis of MSCs revealed that the stiffness of Dex‐TA tethered to cells played an essential role in their lineage commitment. Specifically, single‐cell‐resolution analysis of MSC differentiation revealed that the adipogenic population fraction inversely correlated with microgel stiffness (Figure 2q), while the osteogenic population fraction was characterized by a positive correlation with microgel stiffness (Figure 2r). It is of note, that the single‐cell‐microgel platform offered unique opportunities toward handling, manipulation, and analysis of cells. For example, single‐cell‐microgel analysis revealed the heterogeneity in lineage commitment within a population of differentiating stem cells under controlled chemical and mechanical stimuli. Furthermore, the single‐cell microgels were demonstrated to be compatible with label‐free analyses, which offered the opportunity of time‐lapse monitoring of cell behavior in 3D while minimizing methodology‐induced biases by omitting destructive analyses techniques. Regardless, our data indicated that DOCKING‐mediated tethering facilitated the transduction of biomechanical cues from non‐cell‐adhesive biomaterials to MSC, thereby steering cell fate.

### 2.3 Temporal Stiffening of Tethered Biomaterial Controls Early Onset Stem Cell Lineage Commitment

**Figure 3:**
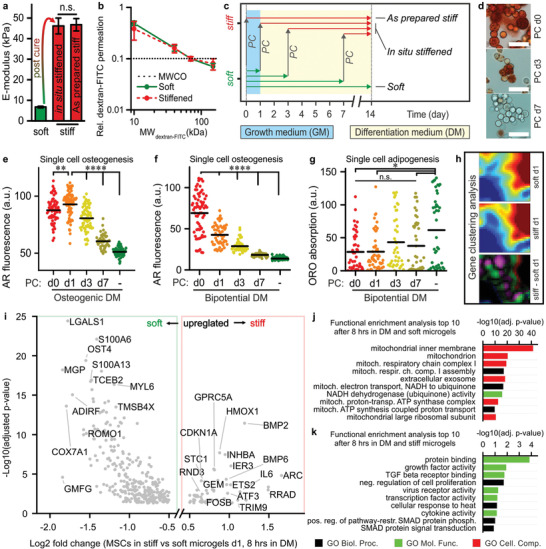
Temporal stiffening of tethered biomaterial controls early onset stem cell lineage commitment. a) Enzymatic post‐curing enabled in situ (i.e., on‐demand) stiffening of soft microgels. The *E*‐modulus of post‐cured (i.e., stiffened) microgels did not significantly differ from as‐prepared stiff microgels. The error bars indicate ± standard error, *n* ≥ 33. Significance is indicated (“n.s.” *p* > 0.05, Mann–Whitney). b) The permeability of stiffened microgels was similar to that of soft microgels, as indicated by comparable MWCOs (dotted line). The error bars indicate ± standard deviation, *n* = 10. c) On‐demand stiffening Dex‐TA microgels on predefined time points via enzymatic post‐curing (PC) was harnessed to study the underlying dynamics of stem cell lineage commitment. d–g) This strategy revealed that short‐term biomechanics steered long‐term cell fate in the presence of osteogenic (d,e) and bipotential (f,g) differentiation medium (DM), where adipogenic and osteogenic differentiation were predisposed to late and early stiffened microgels, respectively. The lines indicate means, *n* ≥ 40 (e), *n* ≥ 50 (f), and *n* ≥ 40 (g); significance is indicated (^****^ *p* < 0.0001, ^**^ *p* < 0.01, ^*^ *p* < 0.05, “n.s.” *p* > 0.05, Kruskal–Wallis ANOVA, validated with Mann–Whitney individual sample comparison). h) Gene clustering analysis of sequenced RNA that was differentially expressed in soft versus stiff single‐cell microgels after 8 h culturing in bipotential DM revealed a shift in gene expression. i) A volcano plot of the soft versus stiff differentially expressed (*p* < 0.05) and protein encoding genes, of which a few are tagged. A cut‐off filter for |log2 fold change| > 0.5 was applied. j,k) The genes in the volcano plot upregulated in soft (j) and stiff (k) microgels were analyzed for functional enrichment, which resulted in gene ontology (GO) term lists revealing the biological processes, molecular functions, and cellular components associated with early onset MSC differentiation in soft and stiff microgels. The scale bars indicate 50 µm.

We then created 3D stem cell microniches with in situ tunable stiffness to further investigate the dynamics of biomechanically imposed stem cell lineage commitment within tethered microenvironments. Specifically, we harnessed the remaining free tyramines in soft microgels to enable their on‐demand stiffening using an enzymatic post‐cure. Nanoindentation measurements revealed that enzymatically post‐curing soft microgels for 90 s significantly increased the stiffness of the microgels (from 6.7 ± 0.4 to 46.1 ± 3.9 kPa; **Figure** **3**a). Notably, the *E*‐modulus of on‐demand stiffened microgels was not significantly different from as‐prepared stiff microgels (46.8 ± 3.0 kPa). On‐demand stiffening of microgels did not notably change the polymer's network porosity, indicating that enzymatic post‐curing predominantly altered the microgels’ biomechanical properties, but not their permeability to biochemical compounds (Figure 3b; Figure S17, Supporting Information). The in situ enzymatic stiffening procedure was not detrimental to the viability of MSCs as compared to as‐prepared stiff microenvironments (Figure S18, Supporting Information). Microgel stiffness could not be changed by polymer network degradation as mammalian cells do not produce dextranase^[^ ^30^ ^]^ and Dex‐TA has been proven to be hydrolytically resistant under similar conditions for several months.^[^ ^31^ ^]^ Besides HRP, other enzymes explored for crosslinking phenol‐functionalized hydrogels include laccase, hematin, and tyrosinase.^[^ ^32^ ^]^ Yet, uncontrolled stiffening of implanted Dex‐TAB hydrogels is not expected, because HRP and laccase are non‐mammalian, hematin requires × 10^−3^ m concentrations of H_2_O_2_ for significant crosslinking,^[^ ^32^ ^]^ and native tyrosinase can only effectively crosslink phenolic materials at supraphysiological enzyme concentrations.^[^ ^33^ ^]^ However, we do envision that Dex‐TAB could potentially be stiffened in vivo in a non‐invasive yet controlled manner via a previously reported visible light‐induced crosslinking strategy of phenolic hydroxyl groups using a ruthenium complex and sodium persulfate.^[^ ^34^ ^]^

Delayed stiffening of on‐cell‐tethered Dex‐TA microniches significantly reduced MSCs’ osteogenic differentiation potential (Figure 3c–e). In fact, providing a stiff microenvironment within the first week of culture in osteogenic differentiation medium was essential to induce osteogenic differentiation of MSCs, which indicated the presence of a discrete time window in which MSCs are susceptible to biomechanical cues directing stem cell lineage commitment. To assess the effects of material stiffness‐induced mechanotransduction on stem cell lineage commitment under more physiological conditions,^[^ ^35^ ^]^ we explored timed stiffening of on‐cell‐tethered Dex‐TA in a bipotential (i.e., mixed adipogenic and osteogenic) differentiation medium. Stiffening MSC‐laden Dex‐TA in bipotential differentiation medium on days 0, 1, 3, and 7 also resulted in a gradual and significant decrease in osteogenic differentiation, which was even more pronounced than in osteogenic medium, and also associated with an increase in adipogenic differentiation (Figure 3f,g; Figure S19, Supporting Information). These data corresponded to previous work describing differentiation of MSCs atop a temporally stiffened cell‐adhesive (i.e., RGD‐functionalized) 2D hydrogel system.^[^ ^36^ ^]^ Together, our findings confirmed the pivotal importance of early onset mechanotransduction on stem cell lineage commitment within engineered 3D microniches, which can be achieved via DOCKING‐mediated cell tethering, even in non‐cell‐adhesive biomaterials.

To study early onset lineage commitment of stem cells induced by the mechanical properties of on‐cell tethered hydrogel microniches in an unbiased manner, we sequenced the transcriptome of microencapsulated MSCs 24 h post encapsulation. Specifically, MSCs from three different human bone marrow donors were tethered and cultured in soft and stiff Dex‐TA microgels for 16 h, then cultured for 8 h in bipotential differentiation medium, and subsequently lysed and sequenced (Figure S20, Supporting Information). Single‐cell microgels only cultured in growth medium were used to determine baseline gene expression levels. Clustering analysis of the obtained transcriptomes (21 491 expressed genes detected) revealed that gene expression profiles from MSCs in soft and stiff microgels had already become notably different within 1 day (Figure 3h; Tables S1 and S2, Supporting Information). The transcriptomes were then analyzed based on the relative up‐ and downregulation of gene expressions in soft versus stiff Dex‐TA microniches (Table S3, Supporting Information). The comparative transcriptome was filtered by selecting protein encoding RNA and removing ribosomal RNA (381 genes left), of which a total of 361 genes were up‐ or downregulated with |log2 fold change| > 2 (Figure 3i; Table S4, Supporting Information). The resulting transcriptome represented the early response elements that potentially initiated MSC's early lineage commitment as a result of the on‐cell‐tethered biomaterial's mechanical properties. Notably, cells tethered in soft microgels were enriched in expression of pro‐adipogenic factors including *LGALS1*,^[^ ^37^ ^]^ *MGP*,^[^ ^38^ ^]^ *ADIRF*,^[^ ^39^ ^]^ and *COX7A1*,^[^ ^40^ ^]^ while cells tethered in stiff microgels expressed more pro‐osteogenic factors such as *FOSB*,^[^ ^41^ ^]^ *STC1*,^[^ ^42^ ^]^ *BMP2*, and *BMP6*.^[^ ^43^ ^]^ Gene ontology (GO) enrichment analysis of the differentially expressed genes suggested that early stem cell commitment associates at the genetic level with altered TGFB/SMAD signaling and metabolic (i.e., mitochondrial) behavior (Figure 3j,k; Tables S5 and S6, Supporting Information). Altered metabolic activity was confirmed by quantification of mitochondrial reductase activity, which was 28 ± 8% higher in MSCs tethered within soft microgels as compared to MSCs tethered within stiff microgels (Figure S21, Supporting Information). Previous work has shown that increased mitochondrial activity is a prerequisite for MSC differentiation into adipocytes.^[^ ^44^ ^]^ Together, these results indicated that mechanotransduction induced by on‐cell‐tethered biomaterials guides cell fate potentially via altering gene expression profiles and metabolic reprogramming.

### 2.4 Mechanotransduction in 3D‐Tethered MSCs is Not Dependent on Cell Volume Changes and Spreading

**Figure 4:**
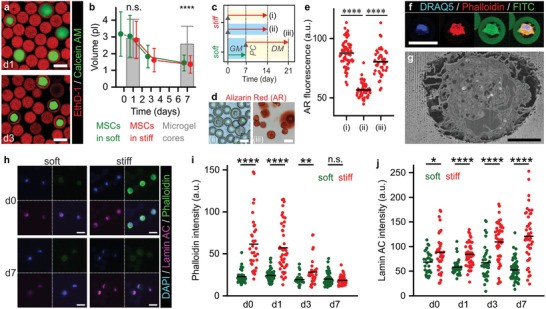
Mechanotransduction in 3D‐tethered MSCs is not dependent on cell volume changes and spreading. a) Confocal microscopic analysis of live/dead stained microencapsulated MSCs showed shrinkage of cells during culture in microgels. b) Time‐lapse quantification of cell volumes revealed that MSCs in both soft and stiff microgels significantly reduced volumes during in vitro culture as compared to the microgel core volume. c) Experimental plan to assess the effect of MSC shrinkage on their osteogenic differentiation potential. d,e) Assessing calcified extracellular matrix using Alizarin Red staining revealed that MSCs cultured for 1 week in soft microgels and GM followed by a stiffening enzymatic post‐cure and 2 weeks culture in osteogenic DM (iii) remained their osteogenic potential, when compared to 2 weeks culture in stiff microgels and osteogenic DM (i), or GM (ii). f) Confocal microscopy and g) FIB/SEM revealed that shrunk cells are still attached to the Dex‐TA microgel interior. Phalloidin staining intensity was artificially boosted. h–j) Time‐lapse confocal imaging of F‐actin (i.e., phalloidin staining) and lamin A/C expression in MSCs in soft and stiff microgels during 1 week of culture in growth medium (GM). The lines in dot plots indicate means, the error bars indicate ± standard deviation, *n* ≥ 29 (b), *n* ≥ 30 (c), *n* ≥ 120 (e), and *n* ≥ 40 (h); significance is indicated (^****^ *p* < 0.0001, ^**^ *p* < 0.01, ^*^ *p* < 0.05, “n.s.” *p* > 0.05, Mann–Whitney). The white scale bars indicate 25 µm and the black scale bar indicates 5 µm.

Stem cell fate and mechanotransduction in cell‐adhesive materials have recently been linked to cell volume changes.^[^ ^28^, ^45^, ^46^ ^]^ We investigated if cell volume also played an important role in the stiffness‐induced programming of stem cells tethered within 3D hydrogel microniches. Specifically, MSCs tethered in Dex‐TA microniches were cultured for 7 days in growth medium, which revealed significant cell shrinkage during the first week after encapsulation (**Figure** **4**a,b; Figure S22, Supporting Information). No clear difference was observed between volume reductions of cells tethered in soft Dex‐TA microniches versus those tethered in stiff Dex‐TA microniches. Unlike previous reports on cell‐adhesive materials, the volume reduction of cells tethered within non‐cell‐adhesive materials was not dependent on material stiffness.^[^ ^45^, ^46^ ^]^

To investigate whether shrinkage of 3D tethered MSCs had an effect on their osteogenic differentiation potential, MSCs tethered within soft microgels were allowed to shrink for 7 days, after which part of the samples were in situ stiffened and exposed to osteogenic differentiation medium for 2 weeks (Figure 4c). Osteogenic differentiation of MSCs that were pre‐incubated and shrunk for 7 days was not more prominent than in MSCs that were immediately differentiated upon tethering within stiff microniches (Figure 4d,e). This indicated that for 3D‐tethered MSCs within a non‐cell‐adhesive material, cell volume was not correlated to osteogenic potential, which contrasts previous work on conventional cell‐adhesive materials.^[^ ^28^, ^45^, ^46^ ^]^

The fate of stem cells has also been correlated to the stress relaxation properties of materials rather than their elastic modulus.^[^ ^47^ ^]^ Such material stress relaxation properties are intrinsically present in native tissues and generally observed in remodelable materials.^[^ ^48^ ^]^ To study the behavior of cells tethered within a remodelable material, we functionalized hyaluronic acid with tyramine (HA‐TA),^[^ ^31^ ^]^ and used DOCKING to tether a mixture of Dex‐TA and HA‐TA (i.e., Dex‐HA‐TA) onto MSCs. Within 1 week of culturing, the volume of MSCs in soft Dex‐HA‐TA significantly reduced to levels similar to MSCs in Dex‐TA (Figure S23, Supporting Information). However, the volume of MSCs tethered within stiff Dex‐HA‐TA did not change during that period. The osteogenic potential of MSCs in soft versus stiff Dex‐HA‐TA was also not significantly different (Figure S24, Supporting Information), corroborating the recently reported positive correlation between MSC volume and osteogenic potential within viscoelastic materials.^[^ ^49^ ^]^ Nevertheless, the fact that osteogenic potential of MSCs tethered within a non‐adhesive and non‐remodelable material such as Dex‐TA was not correlated to cell volume changes, implied that material stiffness‐induced stem cell differentiation could occur independent of cell volume changes.

We then investigated whether yes‐associated protein (YAP) was involved in tethering‐mediated mechanotransduction, as YAP is a well‐known mechanotransduction regulator in cell‐adhesive materials approaches, which specifically activates and accumulates in the cell's nucleus upon cell spreading.^[^ ^50^ ^]^ No positive relation was observed between nuclear translocation of YAP and hydrogel stiffness in tethered MSCs (Figure S25, Supporting Information), which corroborated our hypothesis that mechanotransduction within 3D‐tethered non‐cell‐adhesive microenvironments did not depend on cell spreading.

The comparable levels of MSC shrinkage in soft versus stiff Dex‐TA microenvironments furthermore indicated that Dex‐TA was not susceptible to biofouling of autocrinally secreted cell‐adhesive proteinaceous pericellular matrix during the first week after DOCKING. The deposition of cell‐adhesive proteins such as fibronectin would namely have resulted in fully spread cells that completely occupied the microniche.^[^ ^51^ ^]^ Indeed, treating MSCs in stiff Dex‐TA with integrin inhibitor Cilengitide (i.e., soluble cyclic RGD) could not prevent osteogenic differentiation, which showed that mechanotransduction via DOCKING did not require integrin‐mediated interactions that could have been enabled by nascent ECM (Figure S26, Supporting Information). Our work thereby contrasts previous reports on MSCs in artificial microenvironments, which show that, even in absence of cell adhesive moieties, MSCs can remodel their microenvironment via deposition of nascent cell‐adhesive matrix.^[^ ^52^ ^]^ Possibly, DOCKING did not stimulate production of nascent cell‐adhesive matrix, as the enzymatically crosslinked tethers already facilitated sufficient cell–material mechanotransduction. In agreement, MSCs in RGD‐modified materials also do not change behavior as a result of endogenous ECM production.^[^ ^2^ ^]^

The maintained osteogenic differentiation potential of MSCs in Dex‐TA in the presence of soluble RGD‐peptides was surprising,^[^ ^53^ ^]^ and implied that DOCKING enabled mechanotransduction without the need for adhesion via αVβ3 and αvβ5 integrins. In contrast, in conventional 2D cultures on tissue culture plastic or RGD‐modified hydrogel, MSCs could not adhere in the presence of Cilengitide (Figure S27, Supporting Information). These data corroborated that DOCKING works different than conventional RGD‐mediated adhesion. We postulated that the observed stiffness‐imposed stem cell lineage commitment was related to the direct transduction of mechanical cues from the Dex‐TA hydrogel microniches via DOCKING‐induced tethers. Indeed, high‐resolution confocal imaging and FIB/SEM revealed that, even after shrinkage, cells were still locally attached to Dex‐TA microgels (Figure 4f,g). The observed cell–material connections were presumably formed via tyramine‐tyrosine crosslinking during the DOCKING tethering procedure and could explain the transduction of biomechanical cues from the hydrogel to the shrunk cells.

The near‐immediate altered expression of genes encoding lineage specific transcription factors upon microniche stiffening suggested that tethered MSCs responded via a direct physical intracellular mechanotransduction pathway. When cultured atop stiff cell‐adhesive 2D materials, MSCs are known to upregulate F‐actin, which provides the intracellular forces for cell spreading and volume adaptation, and which positively correlates with osteogenic differentiation of MSCs.^[^ ^45^ ^]^ Tethering MSCs within non‐cell‐adhesive Dex‐TA microgels using DOCKING also associated with an immediate significant increase in F‐actin expression in stiff versus soft material, which diminished over the time course of 1 week (Figure 4h,i). The actin cytoskeleton is known to propagate pericellular forces to the nucleus via lamins (i.e., Lamin A/C), which are major components of the nucleoskeleton.^[^ ^54^ ^]^ Indeed, lamin A/C was expressed at significantly higher levels in MSCs tethered within stiff as compared to soft microniches already within hours after encapsulation and this difference became progressively more distinct over time (Figure 4h,j). The relatively rapid and progressive build‐up of lamins by MSCs tethered in stiff Dex‐TA suggested that mechanical cues were transduced from the cytoskeleton to the nucleoskeleton, thereby demonstrating similar adaptive behavior as observed in 2D MSC cultures on mechanically defined cell‐adhesive substrates.^[^ ^55^ ^]^ However, inhibiting actin polymerization and myosin using cytochalasin D and blebbistatin, respectively, surprisingly could not prevent the osteogenic differentiation of MSCs tethered within stiff Dex‐TA (Figure S26, Supporting Information). Yet, Rho kinase (ROCK) pathway inhibition using Y‐27632 did almost completely diminish osteogenic differentiation, particularly during the first day of differentiation. Inhibiting ROCK pathway after 1 or 3 days of differentiation did no longer have a noticeable inhibiting effect on osteogenic differentiation of MSCs. These results suggested that cell–material tethering via DOCKING could induce early onset cell fate decisioning pathways including ROCK that correlated with, but were not dependent on the actomyosin cytoskeleton.

### 2.5 DOCKING Targets the Integrin Adhesome

**Figure 5:**
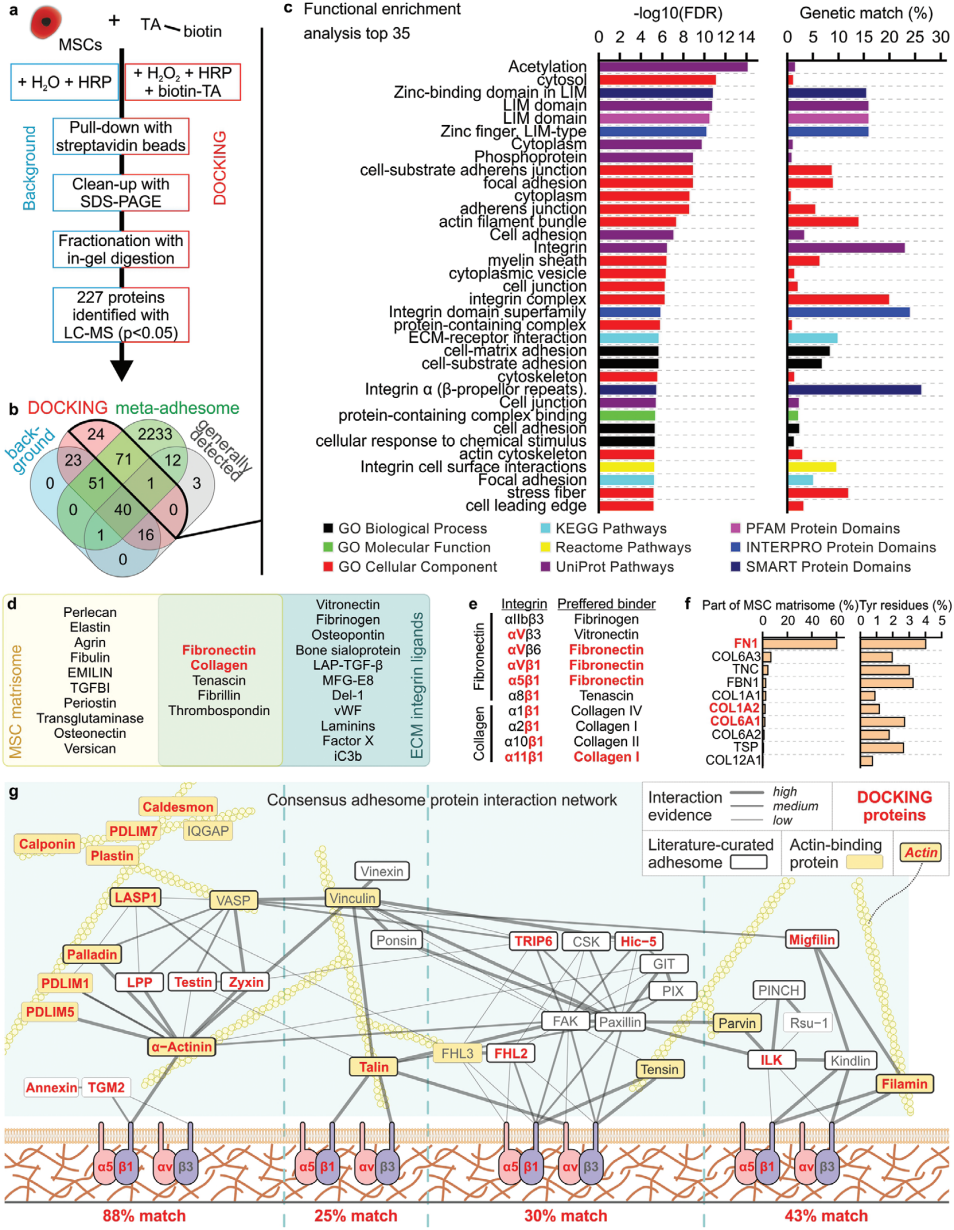
DOCKING targets the cellular integrin adhesome. a) The summarized protocol for isolating and analyzing cellular proteins targeted by DOCKING. b) Using a Venn diagram, the identified proteins were compared to the meta‐adhesome,^[^ ^56^ ^]^ as well as a set of proteins that are generally detected in mass spectrometry.^[^ ^57^ ^]^ c) Functional enrichment analysis top 35 of the proteins identified in the DOCKING sample. d) Venn diagram showing a published MSC ECM proteome (i.e., MSC matrisome)^[^ ^59^ ^]^ and ECM integrin ligands.^[^ ^60^ ^]^ e) Fibronectin and collagen and their binding integrins, as well as the preferred binder of those integrins. f) The relative protein abundance in the MSC matrisome of protein isoforms that have been identified in both the MSC matrisome and reported ECM integrin ligands (i.e., overlap in sub‐panel (d)), followed by a quantification of the percentage of tyrosine residues in those proteins. g) All proteins identified using LC‐MS in the DOCKING sample superimposed on the curated network model of the consensus integrin adhesome protein interaction network. g) Adapted with permission.^[^ ^56^ ^]^ Copyright 2015, Springer Nature. Similar to the original network, interactions were manually validated and scored (high, medium, and low) according to the level of experimental evidence for that interaction, as indicated by the thickness and saturation of the grey edges. The thick black node borders indicate literature‐curated adhesome proteins.^[^ ^71^ ^]^ The yellow node indicates actin‐binding protein. Actin is depicted for illustrative purposes but is not present in the consensus integrin adhesome. Unconnected components or components with only one low‐evidence interaction are not shown in the network. All proteins in (d–f) that were also identified in the DOCKING sample are indicatedwith red text.

To gain insights in how DOCKING‐mediated cell tethering induced cellular mechanotransduction pathways, we determined which cellular proteins were targeted by the enzymatic oxidative crosslinking. To this end, biotin‐tyramide was tethered to MSCs,^[^ ^13^ ^]^ isolated from cell lysates using a pull‐down assay, and analyzed using liquid chromatography combined with mass spectrometry (LC‐MS; **Figure** **5**a). Background protein samples were prepared using the same procedures, but in absence of biotin‐tyramide and H_2_O_2_. A total of 227 proteins were identified, of which 96 proteins were identified as unique targets of DOCKING, 130 proteins were detected in both DOCKING and background samples, and 1 protein was only detected in the background sample (Table S7, Supporting Information). Comparing the identified proteins with the previously reported meta‐adhesome^[^ ^56^ ^]^ as well as a list of generally detected proteins^[^ ^57^ ^]^ revealed that the vast majority (71 out of 96) of DOCKING proteins uniquely associated with the cellular adhesome (Figure 5b; Tables S8–S12, Supporting Information). Functional enrichment analysis of DOCKING target proteins using GO terms revealed a strong association with cell adhesion, and integrin‐based interactions in particular (Figure 5c; Table S13, Supporting Information). Notably, the functional enrichment profile contained several GO terms related to the LIM domain, which is strongly associated with cytoskeletal mechanotransduction.^[^ ^58^ ^]^ A large part (≈43%) of the background proteins overlapped with generally detected proteins consisting of, amongst other, housekeeping genes and commonly observed impurities such as keratins. Background proteins not present in the meta‐adhesome nor generally detected mainly associated with GO terms related to biotin, which was presumably caused by the biotin‐based pull‐down assay (Table S14, Supporting Information).

LC‐MS revealed fibronectin and collagen as the two main ECM targets of DOCKING. Both of these ECM components are part of the MSC matrisome,^[^ ^59^ ^]^ as well as of the list of literature‐curated ECM integrin ligands (Figure 5d).^[^ ^60^ ^]^ From all integrins that have been proven to interact with fibronectin and collagen, integrins αVβ1, α5β1, and α11β1 are among the preferred binders, which were indeed also present in the obtained LC‐MS dataset (Figure 5e). Of the integrin‐binding protein isotypes that both occur in the MSC matrisome and the literature‐curated list of ECM integrin ligands, FN1, COL1A2, and COL6A1 were detected by LC‐MS. Since FN1 is most abundantly present in the MSC matrisome (>60%) and contains the highest percentage of tyrosine residues (4.0%), we postulated that fibronectin acted as the main ECM tethering target of DOCKING (Figure 5f).

To comprehend the molecular mechanisms involved in mechanotransduction following DOCKING‐mediated cell–material tethering, the proteins identified with LC‐MS were superimposed on the consensus integrin adhesome. The consensus integrin adhesome represents the core cell adhesion machinery that is centered around four distinct axes: i) α‐actinin‐zyxin–VASP, ii) talin–vinculin, iii) FAK–paxillin, and iv) ILK‐PINCH–kindlin (Figure 5g).^[^ ^56^ ^]^ We reasoned that mechanotransduction induced by DOCKING was mainly initiated through the direct actin binders (i.e., adapter protein) α‐actinin, talin, and filamin. The α‐actinin‐zyxin–VASP axis had the highest percentage (88%) of matching proteins. Accordingly, α‐actinin has been proven necessary for initial force transmission from the cytoskeleton to adhesion sites, enabling mechanotransduction, reinforcement, and the subsequent maturation of focal adhesions.^[^ ^61^ ^]^ Together, these results indicated that DOCKING‐mediated tethering of cells and materials predominantly transduced mechanical signals to the nucleoskeleton via fibronectin, integrin α5β1, α‐actinin, actin, and various LIM domains.

## 3 Conclusion

We here introduced the concept of discrete inducible on‐cell crosslinking, which was named “DOCKING”. DOCKING enabled the tethering of cells to non‐cell‐adhesive materials via enzyme‐mediated oxidative coupling of phenolic moieties. Particularly, DOCKING facilitated the 3D‐tethering of cells within Dex‐TA and thereby enabled the transduction of cell instructive mechanical cues from the intrinsically non‐cell‐adhesive biomaterial to cells. Furthermore, the non‐cell‐adhesive properties of Dex‐TA were demonstrated to elicit a minimal host response upon implantation as compared to its RGD‐modified version (i.e., Dex‐TA‐RGD). Time‐resolved lineage commitment of 3D‐tethered MSCs was studied by combining DOCKING with temporal tuning of on‐cell tethered material stiffness. This revealed that DOCKING‐mediated mechanotransduction could steer the lineage commitment of stem cells within engineered microniches from an early onset and correlated with F‐actin and Lamin A/C expression. Proteomic analysis indicated that DOCKING targeted the cellular adhesome and in particular fibronectin, presumably enabling mechanotransduction via the α‐actinin axis, and acting independent from cell volume changes and spreading. Uncoupling a material's cell‐adhesive properties from cell binding through discrete inducible cell–material crosslinking uniquely revealed this cell‐volume‐independent behavior; something that has not been observed using materials with intrinsic or extrinsic (e.g., via biofouling) cell‐adhesive properties. In summary, DOCKING provides a unique method to discretely tether cells to molecules or materials, including those that are otherwise non‐cell‐adhesive. This novel method can mitigate chronic inflammation associated with conventional bioligand‐functionalized materials while at the same time providing a cell–material tethering strategy to mechanically program and study (stem) cells in a 3D, temporally controlled, and single‐cell‐resolution manner. As cell–material interactions are instrumental in guiding tissue development, organ homeostasis, disease progression, and repair processes, DOCKING represents a unique tool for optimizing tissue engineering applications, such as regenerative medicine, cultured meat, and organ‐on‐chip platforms.

## 4 Experimental Section

### Materials

Dextran purchased from Sigma/Merck (MW: 15–25 kg mol^−1^; *M*_n_: 16 kg mol^−1^) was functionalized with tyramine, as previously described.^[^ ^19^ ^]^ The resulting Dex‐TA contained ≈15 tyramine moieties per 100 repetitive monosaccharide units. Sodium hyaluronate purchased from Contipro Biotech (MW: 8–15 kg mol^−1^) was functionalized with tyramine, as previously described.^[^ ^31^ ^]^ The resulting HA‐TA contained ≈3 tyramine moieties per 100 repetitive monosaccharide units. 4GRGDSP peptide coupled alginate (alginate‐RGD; Novatach VLVG) was purchased from FMC BioPolymer. HRP (type VI), hydrogen peroxide (H_2_O_2_; with inhibitor), tyramine, tyrosine, fetal bovine serum (FBS), ascorbic acid, iodixanol (OptiPrep), insulin (human), 3‐isobutyl‐1‐methylxanthine (IBMX), indomethacin, dexamethasone, β‐glycerol phosphate disodium salt pentahydrate (β‐GP), Calcein AM, ethidium homodimer‐1 (EthD‐1), thiazolyl blue tetrazolium bromide (MTT), fluorescein isothiocyanate (FITC), dextran‐FITC (10, 40, 70, and 150 kDa), Oil Red O, Alizarin Red S, buffered formalin, dimethyl sulfoxide (DMSO), Triton X‐100, 10‐acetyl‐3,7‐dihydroxyphenoxazine (Amplex Red), dextranase (from Penicillium sp.), sodium acetate, sodium azide, RIPA buffer, protease inhibitor cocktail, phenylmethanesulfonyl fluoride (PMSF), (±)‐6‐hydroxy‐2,5,7,8‐tetramethylchromane‐2‐carboxylic acid (Trolox), TRIS hydrochloride (TRIS‐HCl) ethylenediaminetetraacetic acid (EDTA), biotin, formamide, sodium dodecyl sulfate (SDS), glycerol, bromophenol blue, 2‐amino‐2‐(hydroxymethyl)‐1,3‐propanediol (Trizma base), glycine, acetic acid, methanol, ammonium bicarbonate, dithiothreitol (DTT), iodoacetamide, trifluoroacetic acid (ULC grade), and Aquatex mounting medium were purchased from Sigma Aldrich/Merck. Water (ULC grade), acetonitrile (ACN, ULC grade), and formic acid (ULC grade), were purchased from Biosolve. Urea was purchased from GE Healthcare. Spin filter columns were purchased from Millipore. Cell strainers were purchased from Corning. GentleMACS M Tubes were purchased from Miltenyi Biotec. Tyramide‐AlexaFluor647 (AF647), 4′,6‐diamidino‐2‐phenylindole (DAPI), cell strainers (Falcon), agarose (Ultrapure, low melting point, Invitrogen), Toluidine Blue (Fluka), TRIzol, linear acrylamide, PrestoBlue, bicinchonic acid (BCA) protein assay kit (Pierce), biotinylated protein interaction pull‐down kit (Pierce), Coomassie Brilliant Blue G250, Cryomatrix embedding resin (Shandon), Superfrost Plus Gold adhesion slides, reversed phase LC column (Acclaim PepMap 100 C18, 2 µm, 100 Å, 75 µm × 150 mm), C18 trapping column, and Alexa Fluor labeled donkey‐anti‐mouse secondary antibodies (Invitrogen) were purchased from Thermo Fisher Scientific. Mouse anti‐YAP1 (H00010413) was purchased from Abnova. Mouse anti‐Lamin A/C (sc‐7292) was purchased from Santa Cruz Biotechnology. Aqueous mounting medium (Aqua Poly/Mount) was purchased from Polysciences. Phosphate‐buffered saline (PBS) was purchased from Lonza. Mini‐PROTEAN TGX precast gel was purchased from Bio‐Rad. Enzyme mix trypsin/lysC (mass‐spec grade), Reporter Lysis Buffer (E397A), luciferase assay reagent (E1483), and QuantiFluor dsDNA System were purchased from Promega. Recombinant Human/Mouse/Rat BMP‐2 Protein (BMP2; 355‐BM‐010) was purchased from R&D Systems). RNase‐free H_2_O was purchased from Qiagen. Minimal essential medium α with nucleosides (αMEM), Dulbecco's modified Eagle's medium (DMEM), penicillin and streptomycin, GlutaMAX, 2‐mercaptoethanol, and trypsin‐EDTA were purchased from Gibco. Basic fibroblast growth factor (ISOKine bFGF) was purchased from Neuromics. Biotin‐tyramide was purchased from Iris Biotech GmbH. Lipophilic tracers DiI and DiR, and Phalloidin‐AF488 were purchased from Molecular Probes. Cyclo(Arg‐Gly‐Asp‐D‐Tyr‐Lys) (RGD‐Tyr; PCI‐3662‐PI) was purchased from Peptides International. Cilengitide was purchased from Tocris Bioscience. Cytochalasin D, Blebbistatin (±), and Y‐27632 dihydrochloride were purchased from Enzo Life Sciences. Catalase (from bovine liver) was purchased from Wako. Glycol methacrylate (GMA; Technovit 7100) was purchased from Heraus Kulzer. Polydimethylsiloxane (PDMS, Sylgard 184) was purchased from Dow Corning. Aquapel was purchased from Vulcavite. Pico‐Surf 1 in Novec 7500 Engineered Fluid, Pico‐Surf 1 in Fluorinert FC‐40, and Pico‐Break 1 were purchased from Dolomite. Gastight syringes (Hamilton), fluorinated ethylene propylene tubing (FEP, inner diameter 250 µm, DuPont) and connectors were purchased from IDEX Health and Science. Low‐pressure syringe pumps (neMESYS) were purchased from Cetoni. Surfactant‐free fluorocarbon oil (Novec 7500 Engineered Fluid) was kindly provided by the BIOS Lab‐on‐a‐Chip group (University of Twente). Paraformaldehyde in PHEM buffer and poly‐l‐lysine‐coated Thermanox plastic coverslips where kindly provided by the Section Cryo‐EM (University of Utrecht). C2C12‐BRE‐Luc cells were kindly provided by prof. Daniel B. Rifkin.

### Enzyme‐Mediated Oxidative Tyramine‐Tyrosine Crosslinking

To demonstrate enzymatic tyramine‐tyrosine crosslinking, saturated tyramine and tyrosine solutions were prepared by overnight stirring 2.5 g L^−1^ in demineralized H_2_O (dH_2_O) and subsequent filtration (0.22 µm). Saturated solutions were sequentially mixed with 44 U mL^−1^ HRP and 1 g L^−1^ H_2_O_2_ containing dH_2_O in a 1:1 ratio, resulting in final HRP and H_2_O_2_ concentrations of 22 U mL^−1^ and 0.5 g L^−1^, respectively. After 1 h incubation on a roller mixer, the product was filtered using a spin filter column with 3 kDa molecular weight cut‐off (MWCO) to remove the HRP, and analyzed using positive electron spray ionization MS (Waters Micromass). dH_2_O instead of H_2_O_2_ was used as a negative control. Importantly, filtration removed the HRP, as well as most (non‐water‐soluble) oligomerized phenolic compounds.^[^ ^17^ ^]^ Therefore, mainly phenolic dimers were detected.

### Cell Isolation and Expansion

Human MSCs were isolated from fresh bone marrow samples and cultured as previously described.^[^ ^62^ ^]^ The use of patient material was approved by the local ethical committee of the Medisch Spectrum Twente and informed written consent was obtained for all samples (METC\06003). In short, nucleated cells in the bone marrow aspirates were counted, seeded in tissue culture flasks at a density of 500 000 cells cm^−2^ and cultured in MSC proliferation medium, consisting of 10% v/v FBS, 100 U mL^−1^ penicillin and 100 mg L^−1^ streptomycin, 1% v/v GlutaMAX, 0.2 × 10^−3^ m ascorbic acid, and 1 µg L^−1^ bFGF (added fresh) in αMEM. 3T3 cells were seeded at a density of 2850 cells cm^−2^ and cultured in proliferation medium, consisting of 10% v/v FBS, 100 U mL^−1^ penicillin and 100 mg L^−1^ streptomycin, 1% v/v GlutaMAX, and 71 μm 2‐mercaptoethanol (added fresh) in DMEM. C2C12‐BRE‐Luc cells were cultured in C2C12 proliferation medium consisting of 20% v/v FBS, 100 U mL^−1^ penicillin and 100 mg L^−1^ streptomycin, 1% v/v GlutaMAX, and 0.2 × 10^−3^ m ascorbic acid. Cells were cultured under 5% CO2 at 37 °C and medium was replaced two to three times per week. When cells reached near confluence, the cells were detached using 0.25% v/v Trypsin‐EDTA at 37 °C and subsequently sub‐cultured or used for experimentation.

### DOCKING Fluorophores

To demonstrate DOCKING of fluorophores, MSCs and 3T3 cells were labeled with tyramide‐AF647 by incubation with 3 U mL^−1^ HRP and 0.3 g L^−1^ H_2_O_2_ in PBS. dH_2_O instead of H_2_O_2_ was used as a negative control. For fluorescence confocal microscopy (Nikon A1+), samples were permeabilized using 0.1% v/v Triton X‐100 and subsequently stained with 2.5 U mL^−1^ phalloidin‐AF488 and 1 mg L^−1^ DAPI to stain F‐actin and nuclei, respectively. Cross‐sectional fluorescent intensity plots were prepared using ImageJ software.

### Preparation of Hydrogel Disks and Fluorophore/Cell Tethering Analysis

Dex‐TA bulk gel formation was achieved by mixing 100 g L^−1^ Dex‐TA, 3 U mL^−1^ HRP, and 0.3 g L^−1^ H_2_O_2_ in PBS. Gelation was confirmed using the vial tilting method. Hydrogel disks were produced by injection molding the premixed and ice‐cooled components into disk‐shaped PDMS molds with a diameter and thickness of 5 and 2 mm, respectively. Dex‐TA‐RGD hydrogels were formed by adding 2.2 × 10^−3^ m RGD‐Tyr to the hydrogel premix. Hydrogels were gelled at room temperature. To assess the availability of reactive phenolic moieties in gelled Dex‐TA, the hydrogel disks were labeled with tyramide‐AF647 by incubation with 3 U mL^−1^ HRP and 0.3 g L^−1^ H_2_O_2_ in PBS. dH_2_O instead of H_2_O_2_ was used as a negative control. Furthermore, TA‐AF647 was pre‐crosslinked using HRP and H_2_O_2_ and combined with Dex‐TA disks to confirm that fluorophore‐hydrogel tethering worked via chemical crosslinking rather than physical absorption of poly‐TA‐647 onto the hydrogel. Hydrogel disks were visualized using brightfield and fluorescence microscopy (EVOS FL), and fluorophore labeling was quantified using ImageJ software. To assess cell–material tethering, live MSCs stained with 0.25 g L^−1^ lipophilic tracers (DiI and DiR) according to manufacturer's protocol or formalin‐fixated MSCs were seeded atop 2D Dex‐TA hydrogel substrates. Cell–material crosslinking was induced by adding 44 U mL^−1^ HRP and 0.3 g L^−1^ H_2_O_2_. After 90 s, DOCKING was terminated by adding bovine catalase to a final concentration of 6 kU, which immediately consumed all remaining H_2_O_2_ through a competitive enzymatic reaction.^[^ ^63^ ^]^ The substrates were subsequentially washed with PBS. dH_2_O instead of H_2_O_2_ was used as a negative control. Tethering of MSCs onto Dex‐TA versus Dex‐TA‐RGD hydrogel disks was assessed using phase contrast microscopy after seeding 1000 cells cm^−2^. Alginate‐RGD hydrogels were prepared by injecting 1% v/v alginate‐RGD in PBS into a disk‐shaped mold and covering it with 1 × 10^−3^ m ethanol and 5% v/v ethanol in dH_2_O. After overnight gelation, alginate‐RGD disks were washed once and then incubated for 1 h with MSC proliferation medium.

### BMP Reporter Assay

C2C12‐BRE‐Luc were seeded cells at 10 000 cells cm^−2^ on tissue culture plastic and cultured overnight. Cells were starved using starvation medium consisting of C2C12 proliferation medium with only 0.5% v/v FBS for a period of 8 h. Following starvation, cells were washed with PBS and tethered with Dex‐TA by adding a mixture of 3.75 g L^−1^ Dex‐TA and 22 U mL^−1^ HRP in PBS, followed by an equal volume of 0.3 g L^−1^ H_2_O_2_ in PBS. After 90 s, DOCKING was terminated by adding bovine catalase to a final concentration of 6 kU. dH_2_O instead of H_2_O_2_ was used as a negative control. The samples were washed with starvation medium and subsequently exposed to 100 ng mL^−1^ of BMP2 for 15 h. Subsequently, the cells were lysed using reporter lysis buffer and a single freeze–thaw cycle. Luciferase expression was determined using a luciferase assay following manufacturer's protocol and a luminometer (Victor X3, Perkin Elmer). Luciferase expression was normalized to the total DNA content, which was quantified using the QuantiFluor dsDNA System following manufacturer's protocol and a fluorometer (Victor X3).

### Implantation of Hydrogels

The Dutch Central Committee of Animal Experiments and the Animal Welfare Body at the University of Groningen approved all the described animal procedures (AVD1050020185726). The animals were housed at the Central Animal Facility of the University Medical Center Groningen with ad libitum access to water and standard chow. Briefly, the Dex‐TA and Dex‐TA‐RGD disks were subcutaneously implanted in 8‐week‐old male C57BL/6NCrl mice (Charles River) under 2% isoflurane anesthesia. Each mouse received two disks of either Dex‐TA or Dex‐TA‐RGD (*n* = 4 for both groups). After implantation all mice received a single injection with buprenorphine (0.1 mg kg^−1^) as post‐surgery analgesia. Wound healing and their weights were checked on a regular basis. Mice were sacrificed and the hydrogel disks were explanted after 4 weeks.

The explants were fixated with 20 g L^−1^ fresh paraformaldehyde solution and processed for GMA embedding. The GMA‐embedded disks were cut into 2 µm thin sections and stained with 10 g L^−1^ aqueous Toluidine Blue for 10 s. The sections were quickly washed with distilled water, mounted with Aquatex, and covered with coverslips. The stained samples were imaged using a microscopy slide scanner (Nanozoomer, Hamamatsu) and analyzed using the Aperio ImageScope software (Leica Microsystems). The mean thickness of the fibrotic capsule around each disk was measured in micrometers.

The varying cell types in the cellular overgrowth of capsules were blindly quantified by counting cells at least 500 cells in GMA embedded sections. Cell types were identified according to the morphological characteristics of monocytes/macrophages, lymphocytes, granulocytes, fibroblasts, and multinucleated giant cells. Cells were counted exclusively in areas with active inflammation. This method of quantification of cellular overgrowth on microcapsules was validated and compared with immunocytochemistry on frozen sections in a previous study.^[^ ^64^ ^]^

### Microgel Production and Culture

All microfluidic chips were manufactured from PDMS and glass using standard soft lithography techniques. As previously described,^[^ ^21^ ^]^ the droplet generator and H_2_O_2_ diffusion‐based crosslinking chips were fabricated with ≈25 and ≈100 µm high channels, respectively. Aquapel was introduced in the microfluidic chips before usage to ensure channel wall hydrophobicity. Using FEP tubing, microfluidic chips were connected to each other and to gastight syringes, which were controlled by low‐pressure syringe pumps. For DOCKING with Dex‐TA, hydrogel precursor solution contained 10% v/v Dex‐TA, 44 U mL^−1^ HRP, and 8% v/v OptiPrep (i.e., to obtain ρ = 1.05 g L^−1^) in PBS and was emulsified in Novec 7500 Engineered Fluid oil containing 2% w/w Pico‐Surf 1 surfactant at a 1:6 (hydrogel:oil) flow ratio (2 µL min^−1^ hydrogel, 12 µL min^−1^ oil). For DOCKING with Dex‐HA‐TA, hydrogel precursor solution contained 5% v/v Dex‐TA, 5% v/v HA‐TA, 44 U mL^−1^ HRP, and 8% v/v OptiPrep (i.e., to obtain ρ = 1.05 g L^−1^) in PBS and was emulsified in Fluorinert FC‐40 oil containing 2% w/w Pico‐Surf 1 surfactant at a 1:6 (hydrogel:oil) flow ratio (2 µL min^−1^ hydrogel, 12 µL min^−1^ oil). To quantify H_2_O_2_ in the microdroplets, emulsions were broken, immediately diluted 10^5^ times with PBS, and mixed 1:1 with 100 × 10^−6^ m Amplex Red (Sigma Aldrich) and 0.2 U mL^−1^ HRP in PBS. After 30 min incubation at room temperature, fluorescence intensities were measured using a spectrophotometer (Victor X3, ex. 545/10 nm, em. 590/10 nm) and correlated to H_2_O_2_ concentrations using a standard curve. To produce single‐cell‐laden microgels, detached cells (passage 2 to 5) were washed with medium, flown through a 40 µm cell strainer, and suspended in the hydrogel precursor solution at a concentration of 10^7^ cells mL^−1^. The cell‐laden hydrogel precursor solution was loaded into an ice‐cooled gastight syringe where it was gently agitated every 10 min using a 2 mm long Teflon‐coated magnetic stirring bar. The microemulsion was broken by washing three times with surfactant‐free fluorocarbon oil and subsequent supplementation of Pico‐Break 1 in the presence of serum containing proliferation medium. Single‐cell‐laden microgels were cultured in MSC proliferation medium, MSC adipogenic differentiation medium, consisting of 10% v/v FBS, 100 U mL^−1^ Penicillin and 100 mg L^−1^ Streptomycin, 1% v/v GlutaMAX, 0.2 × 10^−3^ m ascorbic acid, 10 mg L^−1^ insulin, 0.5 × 10^−3^ m IBMX, 200 × 10^−6^ m indomethacin, and 1 × 10^−6^ m dexamethasone (added fresh), MSC osteogenic differentiation medium, consisting of 10% v/v FBS, 100 U mL^−1^ Penicillin and 100 mg L^−1^ Streptomycin, 1% v/v GlutaMAX, 0.2 × 10^−3^ m ascorbic acid, 10 × 10^−9^ m dexamethasone (added fresh), and 10 × 10^−3^ m β‐GP (added fresh) in αMEM, or a 1:1 mixture of adipogenic and osteogenic medium, which were refreshed three times per week. As a negative control, encapsulated MSCs were cultured in MSC proliferation medium supplemented with 10 × 10^−9^ m β‐GP. To stiffen microgels, they were incubated 30 min with 44 U mL^−1^ HRP, after which H_2_O_2_ was added to a final concentration of 0.3 g L^−1^. After 90 s, the enzymatic post‐cure was terminated by adding bovine catalase to a final concentration of 6 kU, which immediately consumed all remaining H_2_O_2_ through a competitive enzymatic reaction.^[^ ^63^ ^]^ Function blocking experiments were performed by adding 1 mL L^−1^ inhibitor solution (i.e., 1000× concentrated) to the cell culture medium. Concentrated inhibitor solution consisted of 10 × 10^−3^ m Cilengitide in dH_2_O, 2 × 10^−3^ m Cytochalasin D in DMSO, 50 × 10^−3^ m Blebbistatin in DMSO, 9.2 × 10^−3^ m Y‐27632 in DMSO, or DMSO. Inhibitors were added fresh with every medium change. On‐chip droplets and microgels were visualized using a stereomicroscope set‐up (Nikon SMZ800 equipped with Leica DFC300 FX camera). The position of cells in microdroplets or microgels was analyzed using ImageJ software. Microgels were imaged using phase contrast microscopy and confocal fluorescent microscopy, and the encapsulation and size distributions of live (i.e., Calcium AM positive) cells and microgels were measured using Matlab software. Cells could be retrieved from Dex‐TA microgels by incubating them with 100 U mL^−1^ dextranase for 10 min at 37 °C. Cell spreading after retrieval was assessed by measuring the cell surface area and circularity of time‐lapse phase contrast microphotographs using ImageJ software.

### Production of Cell/Microgel Aggregates

Dex‐TA‐RGD microgels were co‐seeded with MSCs into non‐cell‐adhesive microwell chips that were produced by casting 30 g L^−1^ sterile agarose in dH_2_O on an in‐house fabricated PDMS mold, as previously described.^[^ ^65^ ^]^ In short, MSCs and microgels were homogenously seeded into agarose constructs (1.9 cm^2^) containing ≈3000 microwells (200 × 200 × 200 µm^3^) at a seeding density of 50 units (i.e., cells + gels) per microwell. The modular microtissues were cultured in MSC proliferation medium and visualized using fluorescence (confocal) microscopy.

### Characterization of Microgels

Microgels were mechanically characterized using atomic force microscopy (JPK NanoWizard) combined with inverted optical microscopy (Zeiss Axio Observer Z1). Indentation measurements were performed in PBS using a cantilever (spring constant 0.151 N m^−1^) with a glass colloidal probe (radius = 18.55 µm) attached to the tip. To extract the elastic modulus of the beads from the obtained force‐deformation curves, the data were fitted assuming the Hertz model for the deformation of two spheres in contact. The mathematical expression is given below, with *F* being the applied force, *d* deformation, *E* and *R* the relative Young's modulus and radius, respectively.

(1) $$F=\frac{4}{3}Ed^{\frac{3}{2}}\sqrt{R}$$

*E* and *R* are given as follows, with ν being Poisson's ratio (assumed to equal 0.5 for these samples) and the indices referring to the two spheres in contact.

(2) $$\frac{1}{E}=\frac{1-\nu_{1}^{2}}{E_{1}}+\frac{1-\nu_{2}^{2}}{E_{2}}$$

(3) $$\frac{1}{R}=\frac{1}{R_{1}}+\frac{1}{R_{2}}$$

To analyze permeability, microgels were incubated with FITC‐labeled dextran with molecular weights ranging from 10 to 150 kDa for 6 days, after which the fluorescent intensities across the microgels were measured using confocal fluorescence microscopic imaging (Zeiss LSM 510) and quantified using ImageJ software. A relative permeated intensity of 0.1 was arbitrarily chosen as the MWCO.

The crosslinking homogeneity within and between microgels was assessed by staining with 4 × 10^−6^ m EthD‐1 in PBS, visualization using confocal fluorescence microscopy (Zeiss LSM 510) and fluorescence microscopy (EVOS FL), followed by cross‐sectional intensity quantification using ImageJ software.

### Analysis of Cell Viability and Function

Viability and metabolic activity of cells was analyzed by staining with 2 × 10^−6^ m calcein AM (live), 4 × 10^−6^ m EthD‐1 (dead), and 0.5 g L^−1^ MTT (metabolically active) in PBS and visualization using brightfield and fluorescence microscopy (EVOS FL). Additionally, metabolic activity was measured by incubating cell‐laden microgels with PrestoBlue reagent that was diluted ten times with cell culture medium for 6 h at 37 °C. Fluorescence intensities were measured using a spectrophotometer (Victor X3, ex. 560/10 nm, em. 590/10 nm). For further analyses, cell‐laden microgels were first washed with PBS and fixated using 10% buffered formalin.

Adipogenic differentiation was analyzed by staining samples with a filtered (0.45 µm) 1.8 g L^−1^ Oil Red O in a 2‐propanol/PBS mixture (6:4), visualizing using brightfield microscopy, and quantifying the per‐cell intensity of the inverted blue color channel using ImageJ software. Osteogenic differentiation was analyzed by staining samples with a filtered (0.45 µm) 20 g L^−1^ Alizarin Red S in saline dH_2_O, visualizing using fluorescence microscopy, and quantifying the per‐cell fluorescent intensity using ImageJ software. Label‐free hyperspectral coherent anti‐Stokes Raman scattering and spontaneous Raman microscopy were performed using in‐house build setups, as previously described.^[^ ^66^ ^]^

For general fluorescence analysis of (cell‐laden) microgels, samples were permeabilized using 0.1% v/v Triton X‐100 and subsequently stained with 2.5 U mL^−1^ phalloidin‐AF488, 1 mg L^−1^ DAPI, and 4 × 10^−6^ m EthD‐1 to stain cellular F‐actin, cell nuclei, and crosslinked Dex‐TA polymer, respectively, and subsequently analyzed using confocal microscopy (Nikon A1+). For fluorescent immunohistochemical analysis, (cell‐laden) microgels were first cryo‐sectioned. To this end, microgels were suspended in 10 g L^−1^ agarose, which was dripped onto a cold parafilm‐covered substrate to induce gelation and form microgel‐laden agarose constructs. After 5 h impregnation with Cryomatrix, the constructs were snap‐frozen on the cryotome's (Shandon AS620, Thermo Fisher Scientific) cryobar at −60 °C. 7 µm thick sections were transferred to Superfrost Plus Gold adhesion slides and kept in PBS until staining. Slides were then mounted in a slide rack (Shandon Sequenza, Thermo Fisher Scientific) and washed 1× with 1 mL PBS. Samples were incubated 15 min with 150 µL permeation/blocking solution consisting of 0.1% v/v Triton‐X100 and 30 g L^−1^ BSA in PBS, followed by 45 min incubation with 150 µL blocking solution consisting of 30 g L^−1^ BSA in PBS. Slides were incubated for 1 h at room temperature with 120 µl 1:100 primary antibody in blocking solution, washed 3× with 250 µL blocking solution, incubated for 1 h with 120 µL 1:500 secondary antibody in blocking solution in the dark, followed by 1 × 250 µL blocking solution to wash. Slides were then counterstained for 30 min using 2.5 U mL^−1^ phalloidin‐AF488 in PBS and 15 min using DAPI, followed by 3 × 250 µL PBS to wash. Slides were removed from the slide rack and protected using aqueous mounting medium and cover slides.

### Focused Ion Beam and Scanning Electron Microscopy

For FIB/SEM, cell‐laden microgel samples were fixated by mixing them in a 1:1 ratio with fixative consisting of 40 g L^−1^ paraformaldehyde in 1 g L^−1^ PHEM buffer (pH 6.9). After 15 min incubation at room temperature, the fixation solution was refreshed, incubated for 1 h at room temperature, followed by overnight incubation at 4 °C. The fixative was diluted 4× with PHEM buffer and stored at 4 °C until further experimentation. Single‐cell‐laden microgels were selected by hand‐picking using a mouth micropipette that was normally used for embryo transfer procedures. The selected samples were placed on poly‐l‐lysine‐coated Thermanox plastic coverslips, post‐fixated, dehydrated, infiltrated with resin, and plasticized as previously described in detail.^[^ ^24^ ^]^

### RNA Isolation and Sequencing

To extract total RNA, cell‐laden microgels were transferred to gentleMACS M Tubes, washed two times with PBS and resuspended in 1 mL TRIzol. Samples were homogenized using the “RNA_01” program on a gentleMACS dissociator (Miltenyi Biotec) and immediately frozen and stored at −80 °C until further processing. To enable swift processing and prevent RNA degradation, maximally 12 samples were processed per RNA isolation run. After thawing the lysates on ice, 200 µL chloroform was added and shaken vigorously by hand for 15 s. After 5 min incubation at room temperature (for initial phase separation), samples were centrifuged for 15 min at 12 000 g and 4 °C. The upper aqueous phase (≈500 µL) was transferred to a new 1.5 mL Eppendorf tube on ice and 50 µL cold sodium acetate was added to a final concentration of 0.3 m, after which the samples where briefly vortexed. 3.15 µL linear acrylamide up to a final concentration of 15 mL L^−1^ and 550 µL ice cold 2‐propanol were added and mixed by gentle pipetting. Samples were incubated overnight at −20 °C, thawed on ice, and centrifuged for 30 min at 12 000 g and 4 °C. The supernatant was gently removed using gentle pipetting (without disrupting the pellet), after which the pellet was resuspended in 1 mL ice cold 70% v/v ethanol. The pellet was reformed by centrifuging for 5 min at 7500 g and 4 °C. The supernatant was immediately removed and the pellet was air‐dried for ≈15 min on ice and subsequently dissolved in 40 µL RNase‐free H_2_O.

RNA quality was validated using fragment analyses. Sequencing was performed on an Illumina NextSeq 500 sequencer (14 million reads, single read, 75 bp). After quality control and adapter clipping, reads were aligned with the Ensembl GRCh37.75 human reference genome^[^ ^67^ ^]^ using a short read aligner based on Burrows‐Wheeler Transform. Read counts were normalized for sequencing depth and gene length, and were compared to determine differentially expressed genes between conditions using the DESeq2 package v1.14.1 within the R platform v3.3.0 to determine differentially expressed genes (adjusted *p*‐value < 0.05).

Gene expression profiling was performed using the “gene expression dynamics inspector” version 2.1. GO enrichment analysis and functional annotation clustering was performed using the DAVID Bioinformatics Resources version 6.8.^[^ ^68^ ^]^

### DOCKING Protein Pull‐Down and Identification

MSCs in suspension were biotinylated by mixing 2.5 × 10^6^ cells with 500 × 10^−6^ m biotin‐tyramide, 42.5 U mL^−1^ HRP, and 0.3% g L^−1^ H_2_O_2_ in 1 mL PBS. After 60 s, 1 mL quenching solution consisting of 5 × 10^−3^ m Trolox, 2 × 10^−3^ m ascorbic acid, and 10 × 10^−3^ m sodium azide in PBS was added. The cells were washed 1× with 1 mL quenching solutions and 2× with 1 mL PBS, after which they were centrifuged for 3 min at 300 g to form a pellet and frozen at −80 °C. A negative control (i.e., non‐biotinylated) sample was prepared using the same procedure, but with biotin‐tyramide and dH_2_O instead of H_2_O_2_.

Proteins were isolated by thawing the pellets on ice, adding 300 µL RIPA lysis buffer consisting of 1× protease inhibitor cocktail, 1 × 10^−3^ m PMSF, 10 × 10^−3^ m sodium azide, 2 × 10^−3^ m ascorbic acid, and 5 × 10^−3^ m Trolox in RIPA buffer, and vortexing at high speed for 15 s followed by 5 min incubation on ice and again 15 s vortexing. Lysates were clarified by spinning 15 min at 16 000 g and 4 °C. Total protein amounts were quantified using the BCA protein assay kit and a BSA standard curve according to manufacturer's protocol. Samples were measured using a spectrophotometer microplate reader (Multiskan GO, Thermo Fisher Scientific).

Biotinylated proteins were purified using the biotinylated protein interaction pull‐down kit. For every pull‐down, 50 µL streptavidin‐functionalized bead slurry was loaded onto the column, washed 3× with TBS buffer and 3× with RIPA lysis buffer. Then the column was loaded with 250 µL RIPA lysis buffer and 300 µL clarified cell lysate (i.e., supernatant), followed by 90 min incubation at room temperature on a roller mixer. The column was sequentially washed 2× with RIPA lysis buffer, 1× with 2 m urea in 10 × 10^−3^ m TRIS‐HCl (pH 8), and 2× with RIPA lysis buffer. Proteins where eluted from the column using elution buffer containing 10 × 10^−3^ m EDTA (pH 8.2) + 8 × 10^−3^ m biotin and 95% v/v formamide in dH_2_O. The column was incubated with 100 µL elution buffer at 75 °C for 15 min, eluted, and rinsed with 50 µL elution buffer.

Protein samples were further cleaned up by running them through a TGX precast gel for 8.5 min at 130 V using a loading buffer (4×) consisting of 0.2 m Tris‐HCl (pH 6.4), 76 g L^−1^ SDS, 40% v/v glycerol, 0.5 g L^−1^ bromophenol blue, and 40% v/v 2‐mercaptoethanol (added fresh) in dH_2_O, and a running buffer consisting of 3 g L^−1^ Trizma base, 14.4 g L^−1^ glycine, and 1 g L^−1^ SDS in dH_2_O. After electrophoresis, gels were immediately fixated by incubating them for 1 h at room temperature in 10% v/v acetic acid and 50% v/v methanol in dH_2_O. Gels were stained for 1 h on a shaker at room temperature using a solution containing 1 g L^−1^ Coomassie Brilliant Blue G250, 10% v/v acetic acid, and 50% v/v methanol in dH_2_O, and subsequently de‐stained using 10% v/v acetic acid and 50% v/v methanol in dH_2_O until background was clear. Stained gel bands were cut into pieces of ≈1 × 1 mm^2^ and stored in 2% v/v acetic acid at 4 °C until in‐gel digestion.

For in‐gel digestion, gel pieces were processed on a MassPREP digestion robot (Waters), as previously described.^[^ ^69^ ^]^ A solution of 50 × 10^−3^ m ammonium bicarbonate in 50% v/v ACN was used for destaining. Cysteines were reduced with 10 × 10^−3^ m DTT in 100 × 10^−3^ m ammonium bicarbonate for 30 min followed by alkylation with 55 × 10^−3^ m iodoacetamide in 100 × 10^−3^ m ammonium bicarbonate for 20 min. Spots were washed with 100 × 10^−3^ m ammonium bicarbonate to remove excess reagents and were subsequently dehydrated with 100% ACN. 6 µg L^−1^ trypsin/lysC in 50 × 10^−3^ m ammonium bicarbonate was added to the gel plugs and incubation was performed at 37 °C for 5 h. The peptides were extracted using a mixture of 1% v/v formic acid and 2% v/v ACN, and subsequently using a mixture of 1% v/v formic acid and 50% v/v ACN.

For LC‐MS analyses, peptide separation was performed on a ultra‐high performance LC (UHPLC) system (Ultimate 3000 Rapid Separation UHPLC system; Dionex, Thermo Fisher Scientific) equipped with a reversed phase LC column. Peptide samples were first desalted in an online installed C18 trapping column. Desalted peptides were separated on an analytical column with a 90‐min linear gradient from 5% to 35% ACN with 0.1% formic acid at 300 nL min^−1^ flow rate. The UHPLC system was coupled to a mass spectrometer (Q Exactive HF, Thermo Fisher Scientific). DDA settings were as follows: full MS scan between 350 and 1650 m/z at resolution of 120 000 followed by MS/MS scans of the top 15 most intense ions at a resolution of 15 000.

For protein identification and quantitation, the DDA spectra were analyzed with Proteome Discoverer (PD) version 2.2, as previously described.^[^ ^70^ ^]^ Within the PD software, the search engine Sequest was used with the SwissProt Human database Homo sapiens (SwissProt TaxID = 9606) (v2017‐10‐25). The database search was performed with the following settings: enzyme was trypsin, a maximum of 2 missed cleavages, minimum peptide length of 6, precursor mass tolerance of 10 ppm, fragment mass tolerance of 0.02 Da, dynamic modifications of methionine oxidation, and protein N‐terminus acetylation, static modification of cysteine carbamidomethylation.

### Figure Preparation and Statistics

All schematics and figures, including pseudo‐colored FIB/SEM photographs were prepared using CorelDRAW X7 software. All graphs were prepared using OriginPro software. Linear regression and statistical significance analyses were performed using OriginPro software.

## Conflict of Interest

The authors declare no conflict of interest.

## Author Contributions

Conceptualization by T.K. and J.L. Experimental design by T.K., J.F.C., P.J.D., H.L.O., B.J.H., M.K., and J.L. Experiments performed by T.K., S.H., J.F.C., N.G.A.W., P.J.D., W.L., H.L.O., M.N., A.M.S., and B.J.H. Data interpretation by all authors. Manuscript writing and revisions by T.K., M.K., S.R.S., and J.L. Supervision by T.K., P.J.D., H.L.O., P.V., M.K., S.R.S., and J.L.

## Supporting information

Supporting Information

Supporting Table 1

## Data Availability

The data that support the findings of this study are available from the corresponding author upon reasonable request.
