# Supplementary material for: Tethering Cells via Enzymatic Oxidative Crosslinking Enables Mechanotransduction in Non‐Cell‐Adhesive Materials
Source: Adv Mater. 2021 Sep 3;33(42):2102660. doi: 10.1002/adma.202102660 (PMC8530967; doi:10.1002/adma.202102660)
Supplement: Supplementary file 1 — Supporting Information [file ADMA-33-2102660-s001.pdf]

© 2021 Wiley-VCH GmbH

ADVANCED MATERIALS

Supporting Information

for *Adv. Mater.,* DOI: 10.1002/adma.202102660

Tethering Cells via Enzymatic Oxidative Crosslinking Enables Mechanotransduction in Non-Cell-Adhesive Materials

Tom Kamperman, Sieger Henke, João F. Crispim, Niels G. A. Willemen, Pieter J. Dijkstra, Wooje Lee, Herman L. Offerhaus, Martin Neubauer, Alexandra M. Smink, Paulde Vos, Bart J.de Haan, Marcel Karperien, Su Ryon Shin, and Jeroen Leijten**

Supporting Information

Tethering cells via enzymatic oxidative crosslinking enables mechanotransduction in non-cell-adhesive materials

Tom Kamperman, Sieger Henke, João F. Crispim, Niels G.A. Willemen, Pieter J. Dijkstra, Wooje Lee, Herman L. Offerhaus, Martin Neubauer, Alexandra M. Smink, Paul de Vos, Bart J. de Haan, Marcel Karperien, Su Ryon Shin, and Jeroen Leijten

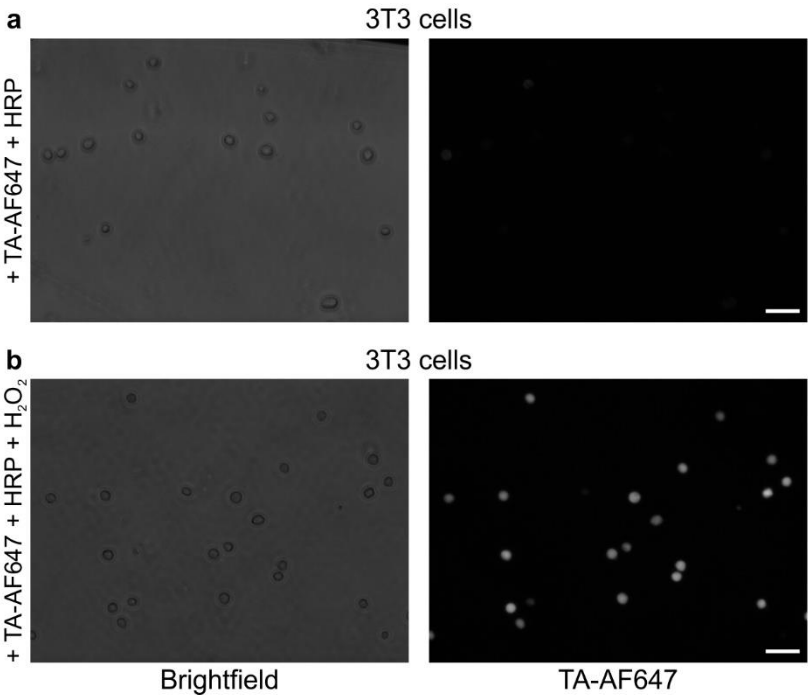

Figure S1. DOCKING of 3T3 cells. (a,b) Enzyme-mediated oxidative crosslinking using HRP and H_2O_2 stained cells with fluorescently labeled tyramine (TA-AF647). Scale bars indicate 50 µm.

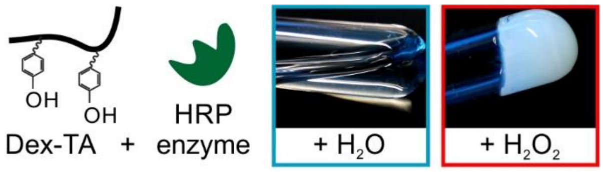

Figure S2. Dex-TA gelation. Enzyme-mediated (using HRP) oxidative crosslinking (using H_2O_2) crosslinking of Dex-TA polymer solution resulted in the formation of Dex-TA hydrogel.

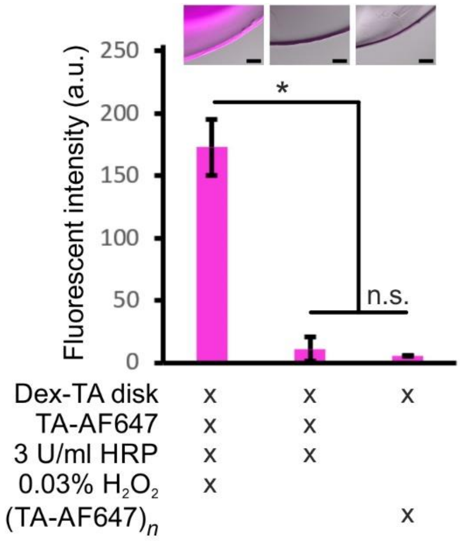

Figure S3. Post-functionalizing preformed Dex-TA disks. Dex-TA disks formed via oxidative crosslinking of tyramine moieties using HRP and H_2O_2 could be further functionalized with tyramine-modified molecules by repeating the enzymatic crosslinking procedure, as demonstrated using fluorescently labeled tyramine (TA-AF647). In contrast, treating Dex-TA disks with TA-AF647 and HRP without performing the enzymatic crosslinking reaction (i.e., without H_2O_2), or with pre-crosslinked TA-AF647 (i.e., (TA-AF647)$_n$), did not result in fluorescent labeling of the preformed Dex-TA disks. Error bars indicate ± standard deviation, n = 3, significance is indicated (* $p < 0.05$, 'n.s.' $p > 0.05$, Mann-Whitney). Scale bars indicate 100 µm.

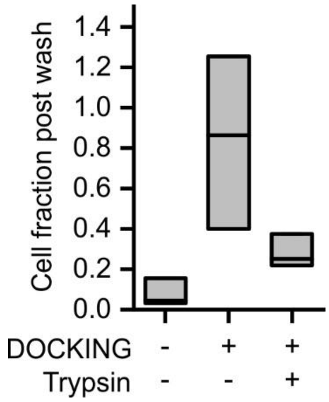

Figure S4. DOCKING adheres cells onto Dex-TA. Quantifying the residual fraction of MSCs atop Dex-TA substrates after washing revealed that DOCKING could trap cells. The cell-material interaction

could be diminished by enzymatic protein digestion using trypsin. Boxes indicate 25-75 percentiles, lines indicate medians, whiskers (overlap with box contours) indicate min-max, *n* = 3.

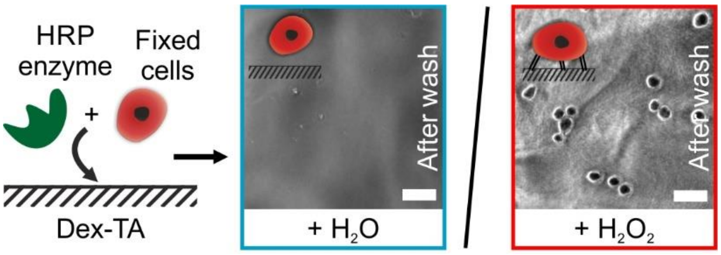

Figure S5. DOCKING adheres fixated cells onto Dex-TA. Formalin-fixated cells seeded atop Dex-TA substrates could be trapped using a HRP-mediated oxidative post-cure (i.e., DOCKING). Scale bars indicate 50 µm.

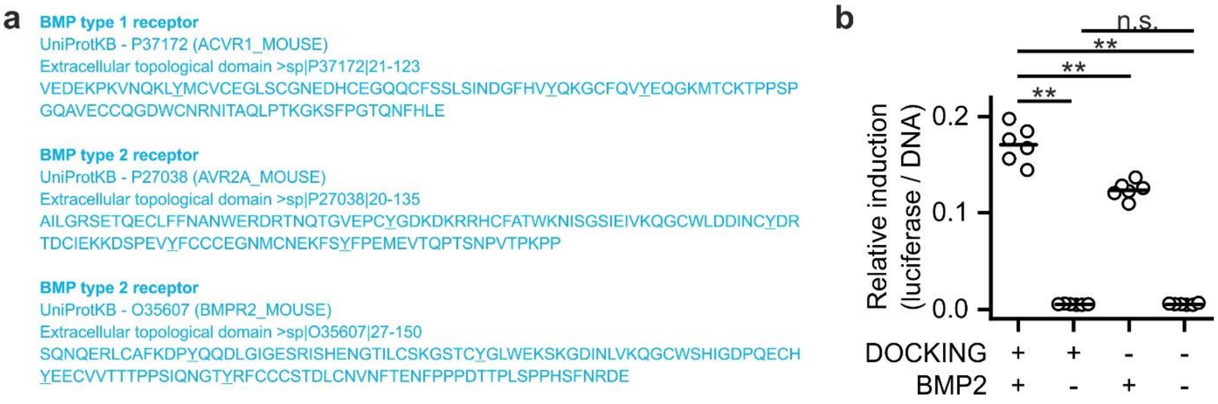

Figure S6. Effect of DOCKING on downstream signaling of BMP receptors in C2C12-BRE-Luc cells. (a) Amino acid sequences of extracellular topological domains of the BMP receptors expressed in C2C12-BRE-Luc cells. Tyrosine residues are indicated with Y. **(b)** The effect of DOCKING Dex-TA onto the downstream canonical signaling of the BMP receptors in the BMP reporter cell line C2C12-BRE-Luc was measured by quantifying the DNA-normalized expression of luciferase upon induction with BMP2. Lines indicate means, *n* = 6, significance is indicated (** $p < 0.01$, 'n.s.' $p > 0.05$, Mann-Whitney).

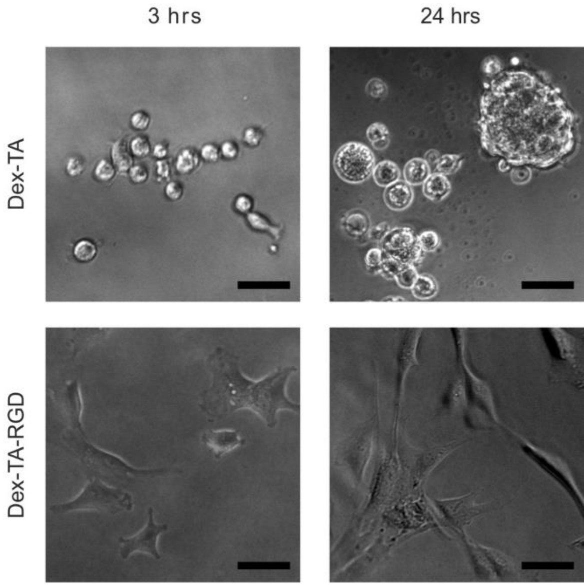

Figure S7. MSC adhesion on Dex-TA versus Dex-TA-RGD. Cells attached and spread onto Dex-TA-RGD, but not Dex-TA after three and 24 hours *in vitro* 2D culture. Scale bars indicate 50 µm.

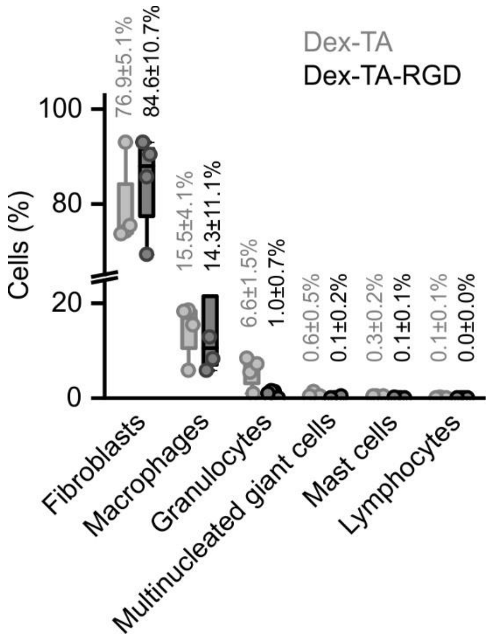

Figure S8. Relative distribution of inflammatory cells around implanted hydrogel constructs after four weeks. At least 500 cells were counted per hydrogel type.

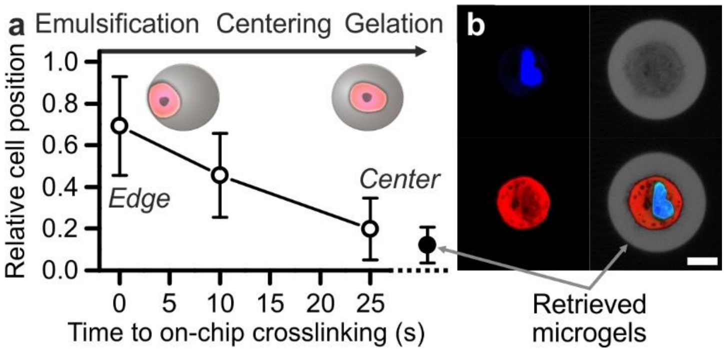

Figure S9. Cell centering in Dex-TA microgels using delayed gelation. (a,b) Delayed gelation enabled centering of the cells within microgels as confirmed using confocal microscopy and previously described.[1] Error bars indicate ± standard deviation, $n \geq 35$. Microgel is stained with FITC, and cell with calcein AM and DAPI. Scale bar indicates 10 µm.

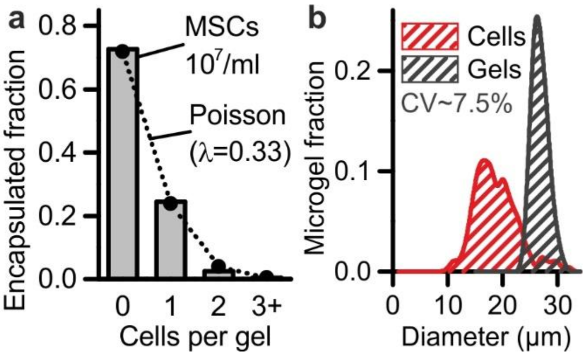

Figure S10. Encapsulation yield and size distributions. (a) The encapsulated cell fraction followed the Poisson distribution. **(b)** The Dex-TA microgels were characterized by a narrow size distribution and were just a few micrometers larger than the cells they encapsulated. The encapsulated cell fraction was determined by analyzing > 1200 microgels. Size distributions were obtained by measuring the diameters of > 250 MSCs and > 500 microgels.

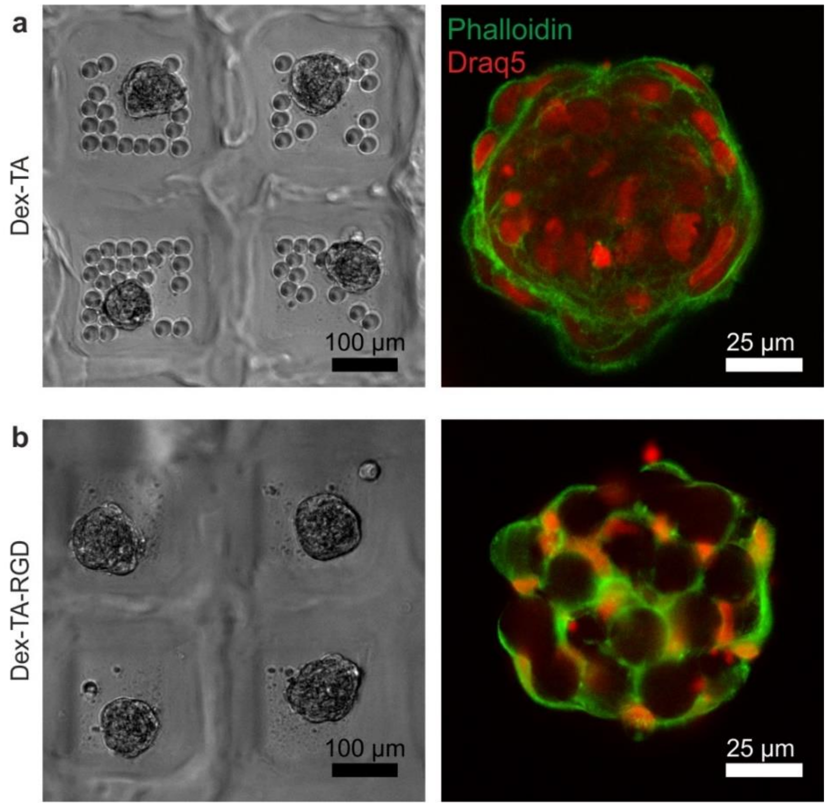

Figure S11. Aggregation of MSCs with Dex-TA versus Dex-TA-RGD microgels. In the absence of DOCKING, MSCs adhered to (non-cell-laden) Dex-TA-RGD but not to (non-cell-laden) Dex-TA microgels. **(a)** Seeding intrinsically non-cell-adhesive Dex-TA microgels with MSCs in non-cell-adhesive agarose microwells resulted in the formation of cell aggregates that did not include the microgels. **(b)** In contrast, culturing MSCs in the presence of Dex-TA-RGD microgels did result in the formation of microaggregates comprising both cells and microgels.

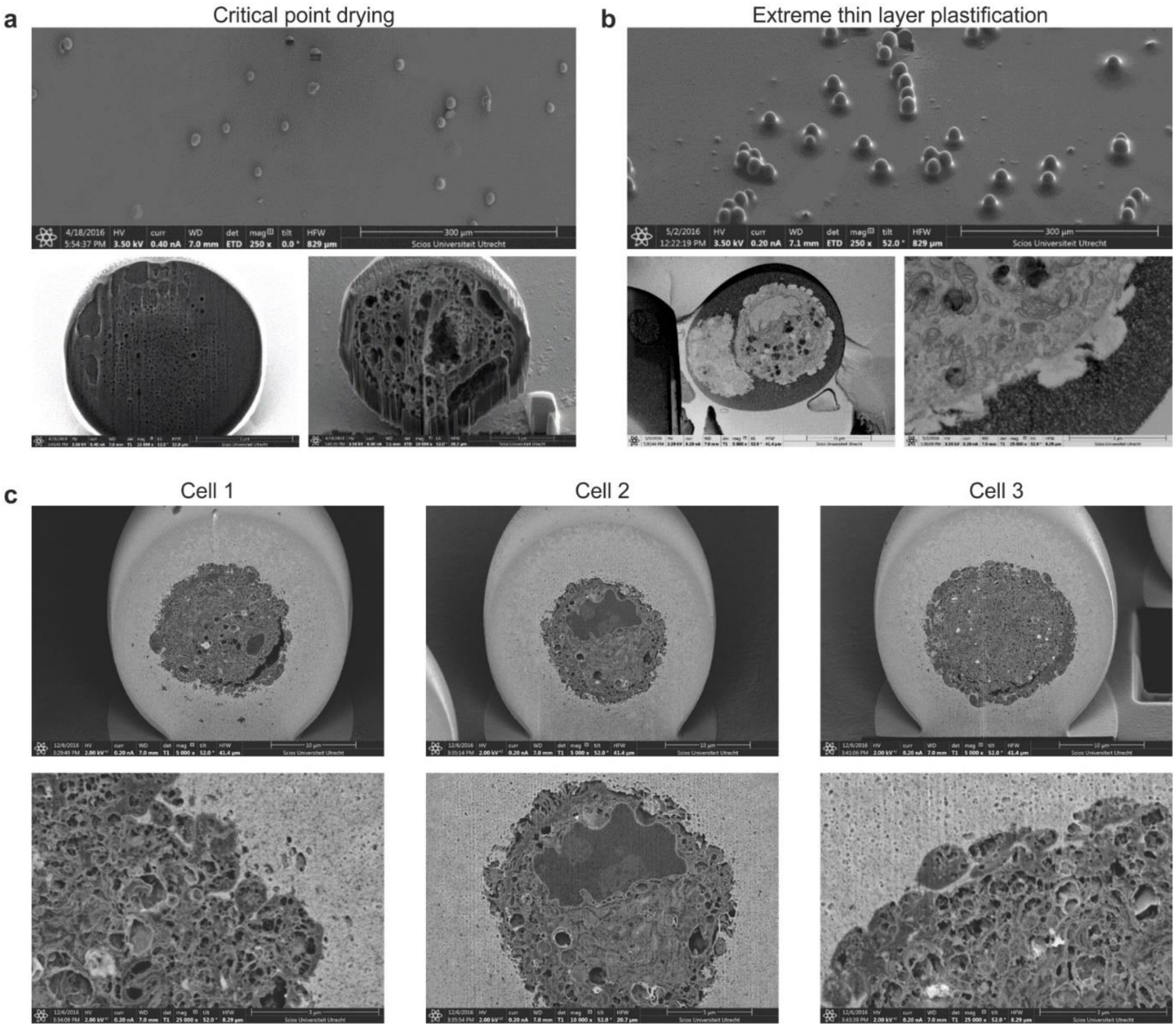

Figure S12. Critical point drying versus extremely thin layer plastification of individual MSCs encapsulated in Dex-TA microgels. (a) The conventional method to prepare samples for SEM analysis resulted in a damaged hydrogel network and hardly detectable intracellular organelles, as also previously observed.[2] **(b)** The extremely thin layer plastification method enabled the high-resolution visualization of single-cell-laden microgels using FIB/SEM.[3] **(c)** FIB/SEM analyses revealed direct contact between the encapsulated cells and the microgel material.

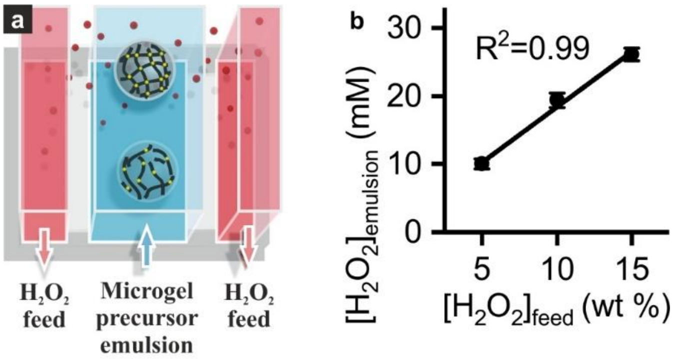

Figure S13. Microfluidic diffusion platform. (a) Schematic depiction of the microfluidic platform consisting of parallel microfluidic channels separated by semipermeable PDMS walls, which enables the diffusion-based supplementation of H_2O_2 into microgel precursor droplets. **(b)** The H_2O_2 concentration within droplets linearly (R^2 = 0.99) correlated to the H_2O_2 feed concentration. Error bars indicate ± standard deviation, *n* = 3.

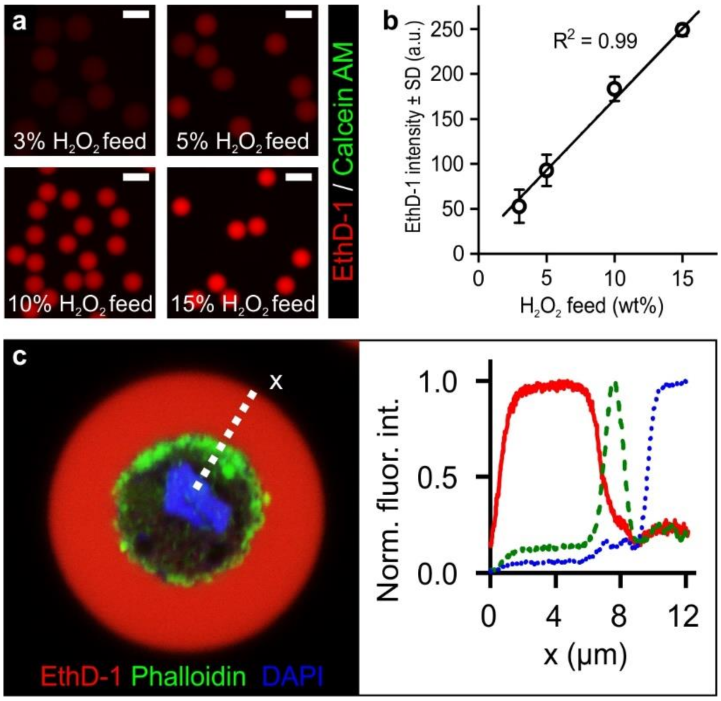

Figure S14. Analyzing Dex-TA crosslinking homogeneity using EthD-1 staining. (a) Dex-TA microgels produced using increasing amounts of H_2O_2 crosslinker resulted in more intense EthD-1 staining, **(b)** which linearly (R^2 = 0.99) correlated to the H_2O_2 feed concentration (i.e., injected in the microfluidic

H_2O_2 diffusion platform). **(c)** Based on the linear relation between Dex-TA crosslinking degree and EthD-1 staining intensity, the intra-microgel crosslinking homogeneity could be analyzed using confocal microscopy. Error bars indicate ± standard deviation, $n \geq 97$. All scale bars indicate 50 µm.

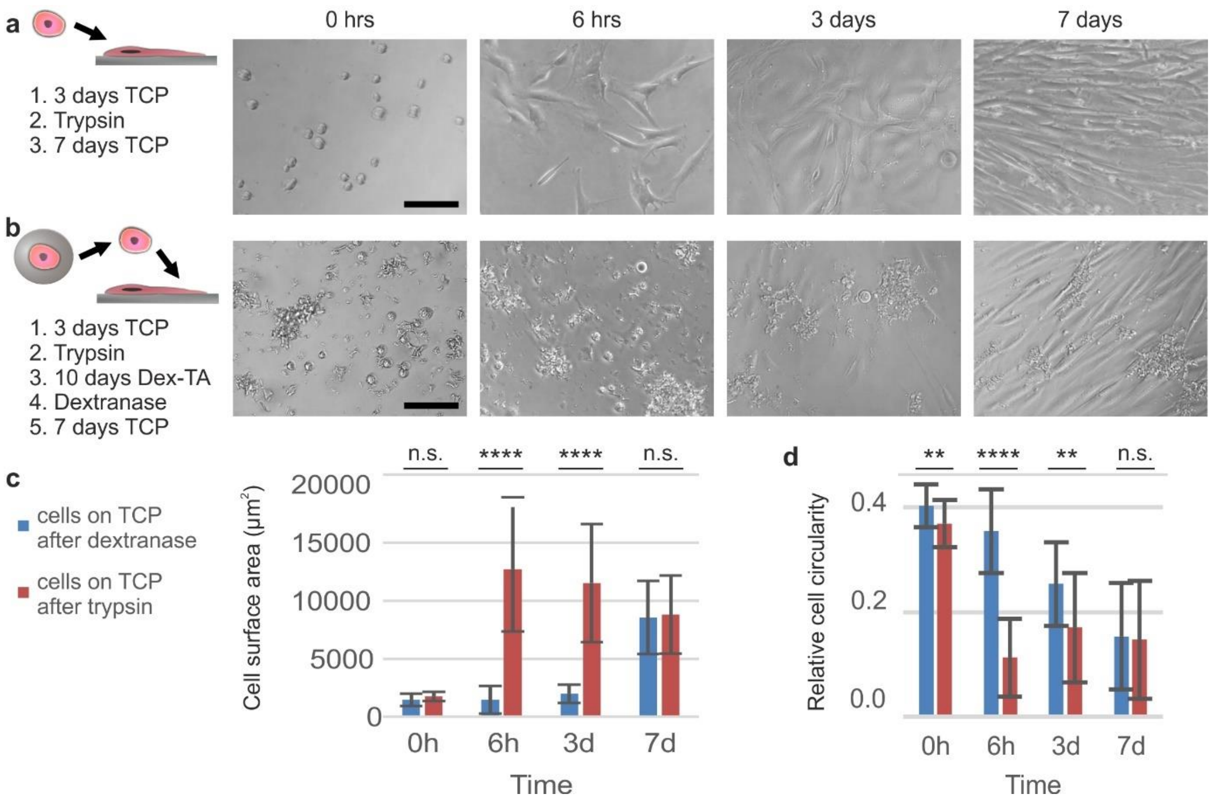

Figure S15. Cell rescue from on-cell tethered Dex-TA microniches using dextranase. (a,b) MSCs were cultured on tissue culture plastic (TCP) for three days, retrieved using trypsin, and then cultured on TCP for seven days (a), or encapsulated in Dex-TA microgels for ten days, retrieved using dextranase, and then cultured on TCP for seven days. **(c,d)** Cell spreading during the second TCP culture was determined by analyzing cell surface area and circularity, which revealed similar results after seven days. Error bars indicate ± standard deviation, $n \geq 15$, significance is indicated (**** $p < 0.0001$, ** $p < 0.01$, * $p < 0.05$, 'n.s.' $p > 0.05$, Mann-Whitney). Scale bars indicate 100 µm.

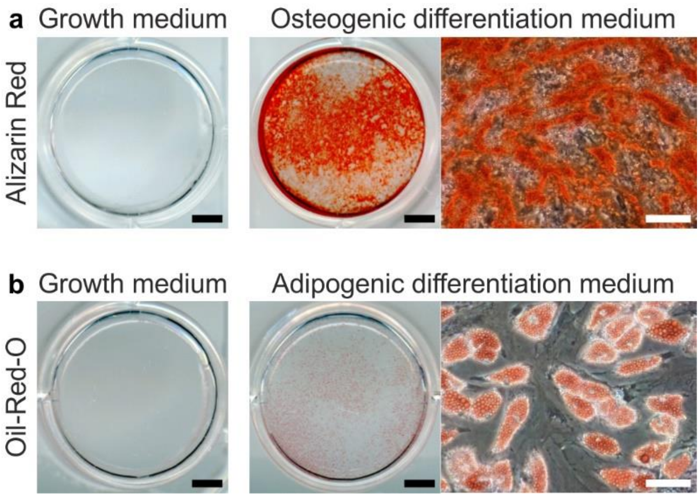

Figure S16. Multilineage differentiation of MSCs in 2D. (a) MSCs cultured on TCP plus osteogenic DM differentiated into the osteogenic lineage within three weeks as confirmed by positive Alizarin Red staining. **(b)** Culturing MSCs in the presence of adipogenic DM resulted in adipogenic differentiation as indicated by positive Oil-Red-O staining. Black scale bars indicate 300 µm, white scale bars indicate 30 µm.

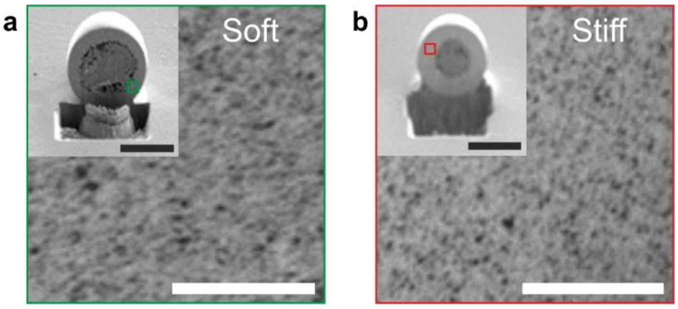

Figure S17. Soft versus stiff microgel porosity. (a,b) FIB/SEM was used to visualize the porosity of soft (a) and stiff (b) single cell-laden microgels. Black scale bars indicate 20 µm, white scale bars indicate 1 µm.

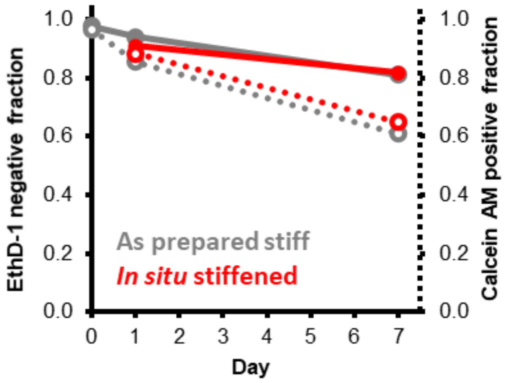

Figure S18. Viability of MSCs in as-prepared stiff versus *in situ* stiffened Dex-TA microniches. EthD-1 negative (i.e., live) cell fractions are indicated with solid datapoints and lines. Calcein AM positive (i.e., metabolically active) cell fractions are indicated with open and dotted datapoints and lines, respectively. Datapoints indicate average, $n \gtrsim 100$.

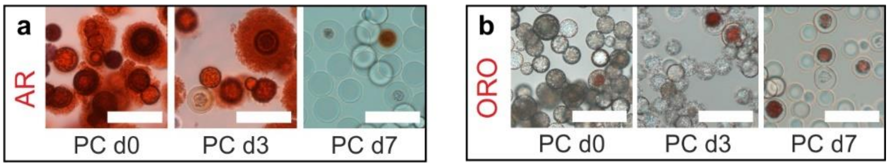

Figure S19. Multilineage differentiation of MSCs encapsulated in soft Dex-TA microgels stiffened at various time points. After two weeks of *in vitro* culture in bipotential DM, **(a)** osteogenic and **(b)** adipogenic differentiation was confirmed by positive Alizarin Red (AR) and Oil-Red-O (ORO) staining, respectively. All scale bars indicate 50 µm.

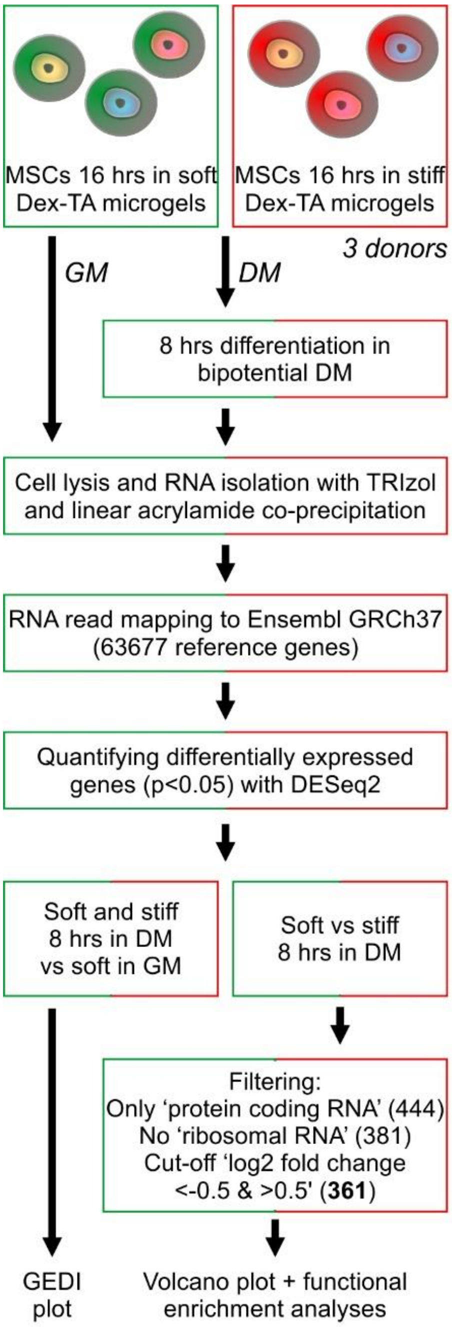

Figure S20. RNA sequencing and analysis protocol.

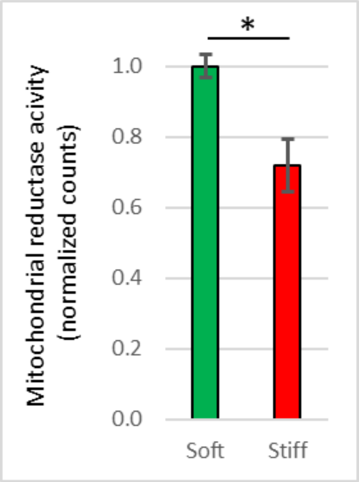

Figure S21. Mitochondrial reductase activity of MSCs in soft versus stiff microgels after one day of culturing. Error bars indicate ± standard error, *n* = 3, significance is indicated (* $p < 0.05$; Mann-Whitney).

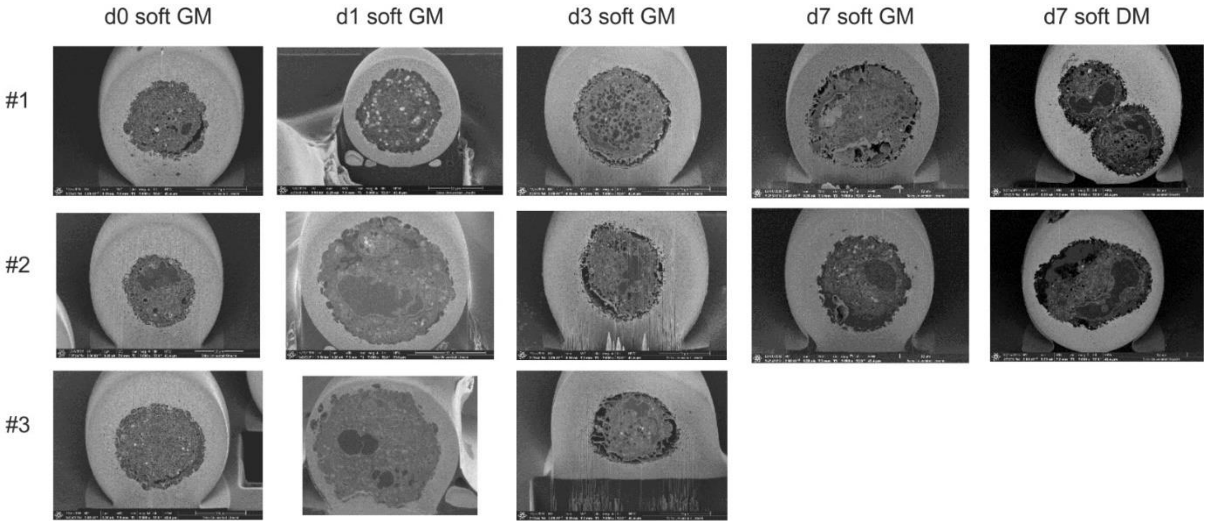

Figure S22. Cell size adaptations in Dex-TA microgels. FIB/SEM analyses revealed that MSCs in both soft and stiff Dex-TA microgels shrink during 3D culture, while cells remain locally connected to the microgel interior.

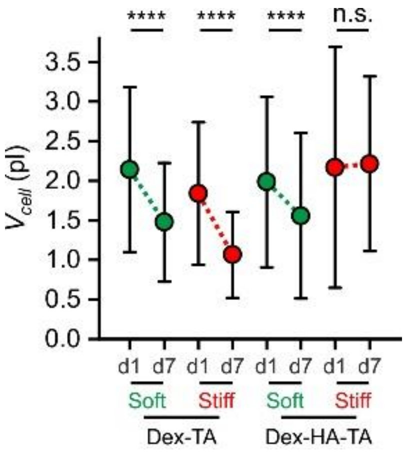

Figure S23. Volume of MSCs tethered within Dex-TA and Dex-HA-TA microniches. Datapoints indicate means, error bars indicate ± standard deviation, $n \geq 47$, significance is indicated (**** p < 0.0001, 'n.s.' p > 0.05, Mann-Whitney).

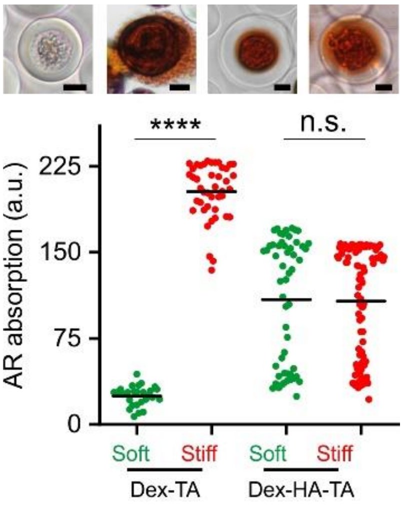

Figure S24. Quantification of the per-cell osteogenic differentiation of MSCs tethered within soft and stiff Dex-TA and Dex-HA-TA microniches. Cells were differentiated in osteogenic differentiation medium for four weeks. Lines in dot plots indicate means, $n \geq 29$, significance is indicated (**** p < 0.0001, 'n.s.' p > 0.05, Mann-Whitney). Scale bars indicate 10 µm.

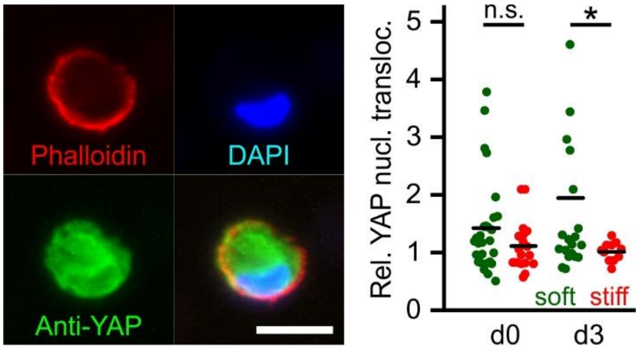

Figure S25. Relative nuclear translocation of YAP in soft versus stiff Dex-TA microgels during culture. Lines in dot plots indicate means, $n \geq 11$, significance is indicated (* $p < 0.05$, 'n.s.' $p > 0.05$, Mann-Whitney).

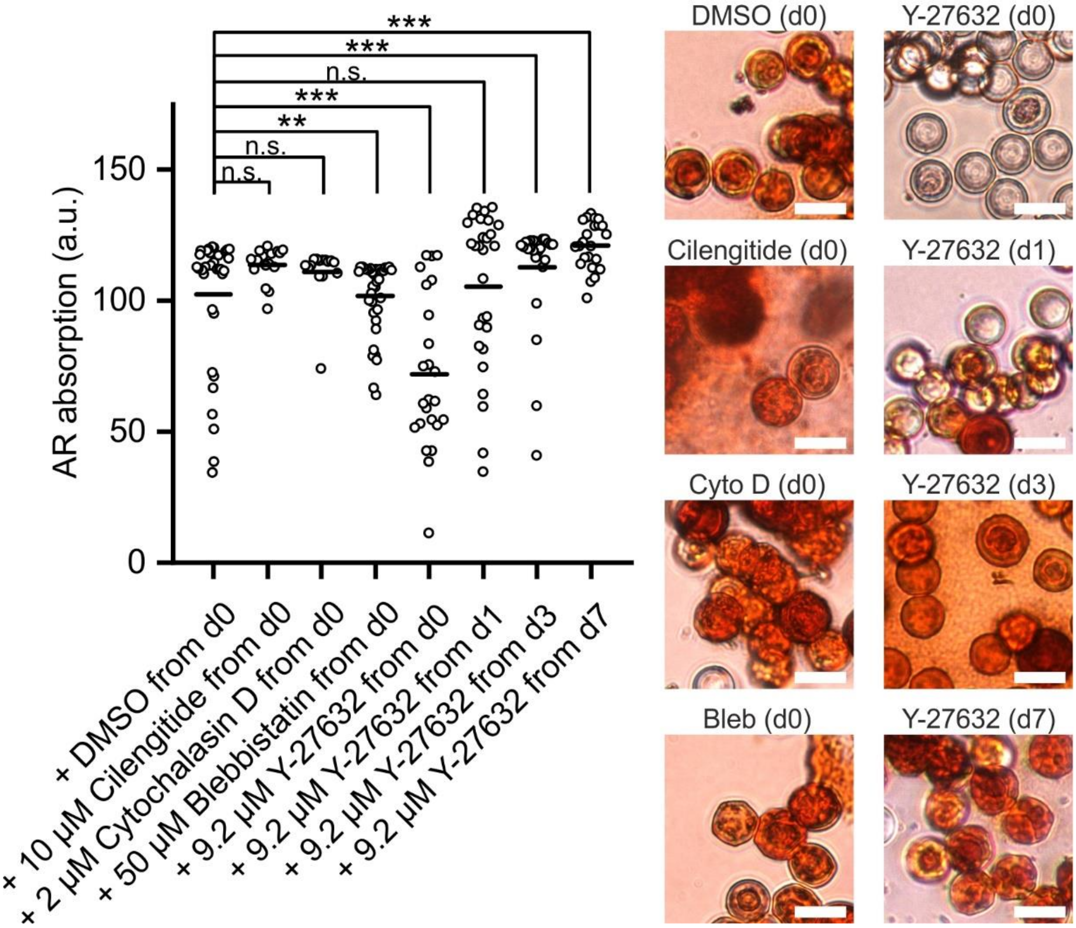

Figure S26. Effect of inhibitors on osteogenic differentiation MSCs 3D-tethered in stiff Dex-TA microniches. Cells were differentiated in osteogenic differentiation medium for three weeks while

treated with different inhibitors from various timepoints as indicated. Some inhibitors needed to be dissolved in dimethyl sulfoxide (DMSO), which was therefore used as control. Lines in dot plots indicate means, $n \geq 15$, significance is indicated (*** $p < 0.001$, ** $p < 0.01$, 'n.s.' $p > 0.05$, Mann-Whitney). Scale bars indicate 50 µm.

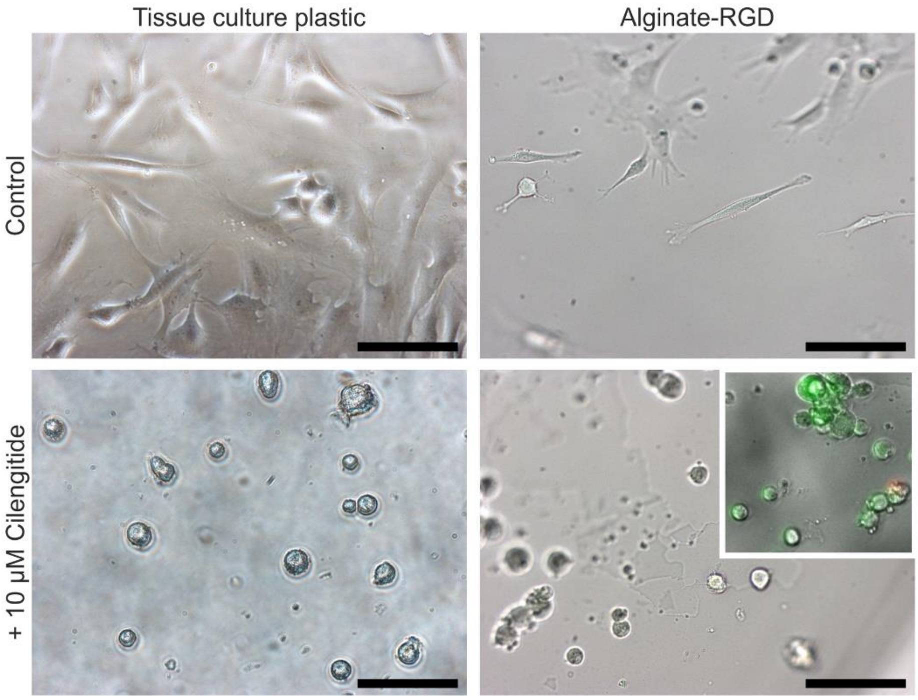

Figure S27. Effect of Cilengitide on adhesion of MSCs in 2D. Before adding Cilengitide, cells were cultured overnight atop tissue culture plastic and alginate-RGD disks to let them adhere and spread. Cilengitide treatment released cells from the surface within minutes and had no noticeable effect on cell survival as shown by live/dead staining (insert). Scale bars indicate 100 µm.

References

[1] T. Kamperman, S. Henke, C. W. Visser, M. Karperien, J. Leijten, Small 2017, 13, 1603711.
[2] T. Kamperman, S. Henke, A. van den Berg, S. R. Shin, A. Tamayol, A. Khademhosseini, M. Karperien, J. Leijten, Advanced healthcare materials 2017, 6, 1600913.
[3] V. A. N. D. EG, B. Dorresteijn, D. Popov-Celeketic, V. D. W. WJ, T. C. Verrips, T. Boekhout, C. Schneijdenberg, A. T. Xenaki, V. D. K. TP, W. H. Muller, J Microsc 2018, 270, 359.
